# Spatiotemporally Precise Rheumatoid Arthritis Therapy via a Triple‐Locked Cascaded Nanoplatform With Dual‐Channel Self‐Reporting

**DOI:** 10.1002/advs.78405

**Published:** 2026-10-22

**Authors:** Jihong Liu, Wendi Zhu, Shiyun Xu, Shichang Liu, Wenjun Tang, Xiaoyu Ma, Ting Yang, Haojian Yang, Hongtong Wang, Lu Cao, Lijie Tan, Qi Wang

**Affiliations:** ^1^ Key Laboratory For Advanced Materials and Joint International Research Laboratory of Precision Chemistry and Molecular Engineering Shanghai Key Laboratory of Functional Materials Chemistry Feringa Nobel Prize Scientist Joint Research Center Institute of Fine Chemicals Frontiers Science Center For Materiobiology and Dynamic Chemistry School of Chemistry and Molecular Engineering East China University of Science and Technology Shanghai China; ^2^ Center of Photosensitive Chemicals Engineering East China University of Science and Technology Shanghai China; ^3^ Department of Thoracic Surgery Zhongshan Hospital Fudan University Shanghai China; ^4^ Department of Orthopedic Surgery Zhongshan Hospital Fudan University Shanghai China

**Keywords:** dual‐channel, fluorescence imaging, orthogonal activation, precision theranostics, rheumatoid arthritis, triple‐locked

## Abstract

Achieving precise spatiotemporal control over combination therapies in rheumatoid arthritis (RA) remains heavily compromised by conventional nanoplatforms, which typically suffer from premature off‐target leakage, coupled drug release, and inadequate in vivo tracking capabilities. Herein, a programmable orthogonally branched triple‐locked nanotheranostic platform (MB@YW NPs) is engineered to seamlessly synchronize spatial localization, chemically orthogonal multidrug liberation, and real‐time multichannel self‐reporting. The system comprises a pH‐responsive polymeric matrix co‐encapsulating a HOCl‐activatable methotrexate prodrug (MB‐MTX) and an ONOO^−^/light‐responsive sulfasalazine prodrug (YW‐SSZ). Precluding systemic leakage, synovial acidity dictates initial spatial targeting by triggering tissue‐specific disassembly and subsequent macrophage internalization. Subsequently, non‐interfering pathophysiological triggers exclusively liberate distinct therapeutics through independent pathways: intracellular HOCl purely unlocks methotrexate with concomitant near‐infrared “turn‐on” fluorescence, whereas an isolated ONOO^−^/light sequence locally activates sulfasalazine accompanied by a blue “on‐to‐off” readout. This lack of cross‐talk successfully discriminates multiplexed activation states. In vivo, this triple‐locked platform efficiently alleviates oxidative stress and remodels the inflammatory microenvironment, leading to markedly suppressed joint destruction in collagen‐induced arthritis models. Furthermore, transcriptomic analysis reveals these synergistic macroscopic benefits are driven by targeted modulation of the cAMP/NF‐κB signaling axis. Collectively, this work establishes a transformative and generalizable framework for independently coordinated, next‐generation precision multi‐prodrug nanomedicine.

## Graphical abstract

An orthogonally programmable nanotheranostic (MB@YW NPs) enables precision therapy for rheumatoid arthritis. A triple‐locked (pH, HOCl/ONOO^−^, light) independently drives the release of methotrexate and sulfasalazine. Trackable by dual‐channel fluorescence, this coordinated drug delivery remodels the inflammatory microenvironment to markedly suppress synovial inflammation and joint destruction in collagen‐induced arthritis models.

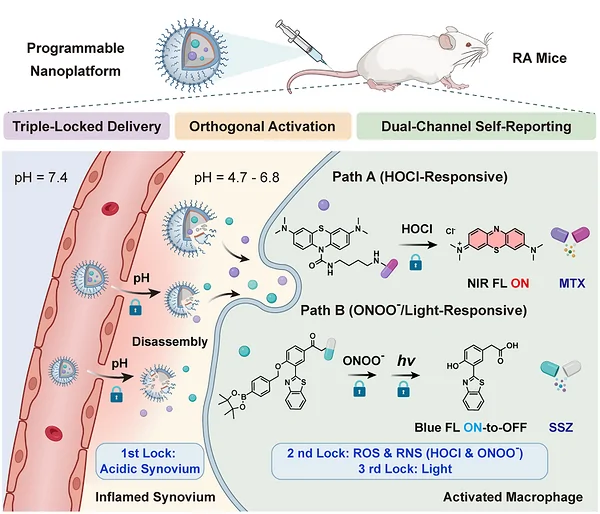

## 1 Introduction

Rheumatoid arthritis (RA) is an autoimmune disorder driven by persistent synovial inflammation and progressive joint erosion [1, 2]. A major driving force in its pathological progression is the vicious cycle between excessive local reactive oxygen species (ROS) and pro‐inflammatory macrophage polarization, which synergistically sustain synovial damage [3, 4]. Clinically, combination regimens based on disease‐modifying antirheumatic drugs (DMARDs), particularly methotrexate (MTX) as a first‐line anchor drug and sulfasalazine (SSZ) as a complementary anti‐inflammatory and immunomodulatory agent, remain a cornerstone of RA management [5, 6, 7, 8]. However, the therapeutic synergy between MTX and SSZ is substantially limited by nonspecific systemic distribution and mismatched pharmacokinetic profiles, which prevent their concomitant accumulation and spatial co‐localization at inflamed joints, thereby compromising therapeutic outcomes and increasing off‐target toxicity [9, 10, 11, 12].

Smart drug delivery systems have improved therapeutic specificity, yet achieving independent spatiotemporal control over multiple cargos remains a major hurdle [13, 14, 15, 16, 17, 18, 19, 20, 21, 22, 23, 24, 25, 26, 27]. When co‐delivering mechanistically distinct agents such as MTX and SSZ, realizing this level of control encounters three practical engineering trade‐offs in carrier design. Single‐stimulus nanocarriers frequently suffer from premature cargo leakage in circulation, because the physiological differences between inflamed joints and normal tissues are often insufficient to ensure complete selectivity [28, 29]. Addressing this off‐target release requires hierarchical spatial gating, where the carrier remains stable during systemic transit and disassembles only when encountering sequential extracellular and intracellular cues [30, 31, 32, 33]. Moreover, conventional co‐delivery vehicles typically rely on uniform matrix degradation or identical cleavable linkages, resulting in coupled drug release [34, 35]. Because MTX and SSZ act through distinct cellular targets and require different local concentrations, their therapeutic synergy benefits substantially from chemically orthogonal activation pathways that liberate each drug independently. Finally, monitoring these multi‐stage processes in vivo is impeded by traditional single‐wavelength imaging modalities, which cannot differentiate the distinct activation kinetics of each therapeutic payload to verify successful on‐target release within the joint lesion [36, 37]. Developing an integrated platform that combines spatial co‐localization, orthogonal drug activation, and distinguishable optical reporting therefore provides a rational strategy to optimize precision RA therapy.

To address these limitations, we herein develop a programmable theranostic nanoplatform, MB@YW NPs, featuring an orthogonally branched triple‐locked architecture that enables spatially confined delivery, independent multidrug activation, and dual‐channel self‐reporting for precision RA therapy (Scheme 1). The system is constructed by incorporating a pH‐responsive polymeric matrix (PEG‐PND) co‐encapsulating a HOCl‐cleavable MTX prodrug (MB‐MTX) and an ONOO^−^/light‐responsive SSZ prodrug (YW‐SSZ) (Scheme 1a). Rather than relying on a uniform activation sequence, the delivery process is orchestrated through a shared initial lock followed by payload‐tailored activation pathways within this triple‐locked framework. Spatial targeting is initially governed by synovial acidity as the shared first lock, which triggers nanoparticle disassembly to facilitate joint retention and macrophage internalization while minimizing premature systemic clearance. Following cellular uptake, the platform bifurcates into distinct activation trajectories. In the MTX activation pathway, elevated intracellular HOCl serves as the second lock to selectively cleave MB‐MTX, achieving complete drug liberation accompanied by near‐infrared (NIR) fluorescence turn‐on. Concurrently, SSZ activation is governed by a two‐step intracellular cascade, in which intracellular ONOO^−^ primes YW‐SSZ into the photosensitive intermediate OH‐SSZ with blue fluorescence emission, after which external light acts as the third lock to achieve on‐demand bioactivation with simultaneous blue fluorescence quenching (“On‐to‐Off”). Collectively, this hierarchically locked platform integrates spatial co‐localization, payload‐specific activation kinetics, and real‐time optical tracking within a single nanocarrier (Scheme 1b). Functionally, MB@YW NPs effectively attenuate oxidative stress and restore inflammatory homeostasis in RA‐relevant microenvironments. In a collagen‐induced arthritis (CIA) mouse model, the system significantly suppresses synovial inflammation and prevents joint destruction, outperforming conventional monotherapy. Comprehensive transcriptomic (RNA‐seq) analysis further unveiled that these therapeutic benefits are mechanistically driven by the targeted modulation of the cAMP/NF‐κB signaling axis (Scheme 1c). Collectively, this work establishes a chemically orthogonal nanotheranostic paradigm that resolves the longstanding challenge of independent multidrug programming and provides a generalizable framework for self‐reporting precision theranostics of complex inflammatory diseases.

**SCHEME 1:**
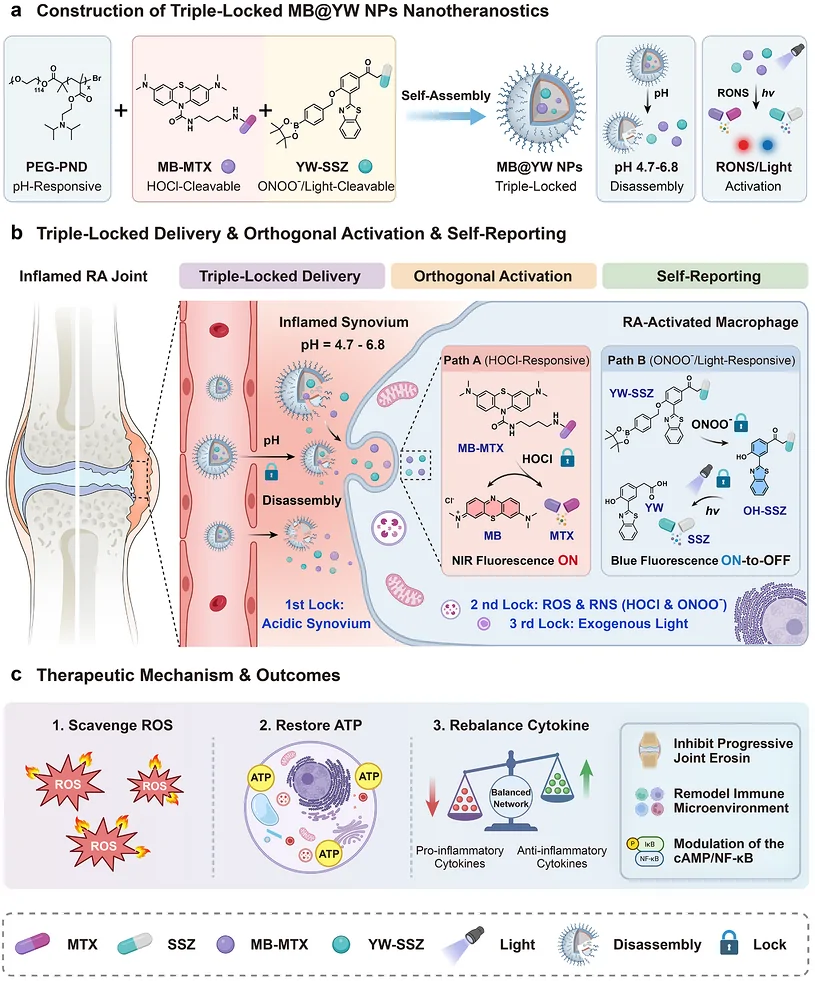
Schematic illustration of the programmable triple‐locked MB@YW nanoplatform for spatiotemporally resolved dual‐channel self‐reporting and orthogonal rheumatoid arthritis therapy. (a) Structural design and composition of MB@YW NPs. The nanoplatform integrates a pH‐responsive polymeric matrix (PEG‐PND) with two chemically orthogonal prodrugs: a HOCl‐cleavable MTX prodrug (MB‐MTX) and an ONOO^−^/light‐activatable SSZ prodrug (YW‐SSZ). This triple‐locked architecture ensures high stability during systemic circulation and prevents premature payload leakage. (b) Orthogonally branched triple‐locked delivery, orthogonal activation, and dual‐channel self‐reporting. Upon accumulation in the inflamed synovium, the first lock is triggered by the acidic microenvironment, enabling nanoparticle disassembly and site‐specific release. Inside inflamed cells, the second lock is orthogonally triggered by distinct pathophysiological cues: elevated HOCl fully liberates active MTX with concomitant NIR fluorescence turn‐on, whereas ONOO^−^ selectively primes YW‐SSZ into the photosensitive intermediate OH‐SSZ with blue fluorescence emission. The third lock is governed by external light irradiation, which further regulates SSZ activation and fluorescence switching. This hierarchical unlocking process enables independently programmable drug activation and real‐time dual‐channel optical readout of therapeutic progression. (c) Proposed therapeutic mechanisms and comprehensive treatment outcomes. The orthogonally actuated therapy effectively scavenges excessive ROS, restores ATP homeostasis, and rebalances the dysregulated cytokine network, ultimately leading to the comprehensive prevention of bone erosion, the remodeling of the immune microenvironment, and the modulation of the cAMP/NF‐κB signaling axis.

## 2 Results and Discussion

### 2.1 Molecular Design and Logic‐Gated Architecture of the MB@YW Nanoplatform

The construction of the programmable MB@YW nanoplatform was predicated on a hierarchical, multi‐stage unlocking logic designed to navigate the complex physiological barriers and biochemical heterogeneity of the RA microenvironment. To prevent premature payload leakage and ensure specific drug activation, the nanoplatform is engineered to undergo a stepwise structural and chemical evolution. This process is strictly controlled by three distinct molecular triggers, defined here as independent “locks”, which coordinately respond to local acidity, intracellular oxidative stress, and targeted photo‐irradiation along payload‐tailored pathways.

The first lock is established by a pH‐responsive amphiphilic copolymer, PEG‐PND, which serves as the protective outer shell of the MB@YW nanoplatform [38]. This intelligent envelope undergoes a sharp hydrophobic‐to‐hydrophilic transition upon encountering the acidic synovium (pH 4.7–6.8) via the protonation of the tertiary amine pendants on the PND segments, triggering the programmed disassembly of the nanostructure (Figure 1a) [39, 40]. This gating mechanism not only ensures robust structural integrity and cargo sequestration during systemic circulation at physiological pH 7.4 but also facilitates the precise, site‐specific liberation of the encapsulated small‐molecule prodrug monomers, thereby enabling enhanced deep‐tissue penetration within the inflamed joint matrix and their subsequent internalization by RA‐activated macrophages. The second lock is governed by the intracellular inflammatory microenvironment, where macrophage‐derived oxidative stress signals (HOCl and ONOO^−^) activate orthogonal prodrug transformation pathways. The HOCl‐responsive MB‐MTX prodrug undergoes selective oxidative cleavage via a tailored urea linkage, leading to MTX release accompanied by NIR fluorescence activation (λ_ex/em_ = 625/699 nm) (Figure 1e) [41, 42]. In parallel, ONOO^−^ triggers oxidative deprotection of the pinacol boronate moiety in YW‐SSZ, generating a phenolic blue‐emitting intermediate (λ_ex/em_ = 375/468 nm). The third lock is provided by external light irradiation, which functions as an on‐demand temporal regulatory gate for the ONOO^−^‐primed YW‐SSZ intermediate. Upon photoirradiation (400–650 nm), the activated intermediate undergoes excited‐state intramolecular proton transfer (ESIPT)‐assisted deprotonation followed by photo‐Favorskii rearrangement, resulting in the release of SSZ as the terminal output (Figure 1i) [43, 44, 45]. This light‐triggered release is accompanied by a transition of the fluorescence signal from “on” to “off”, enabling self‐reporting optical visualization of SSZ photorelease.

**FIGURE 1:**
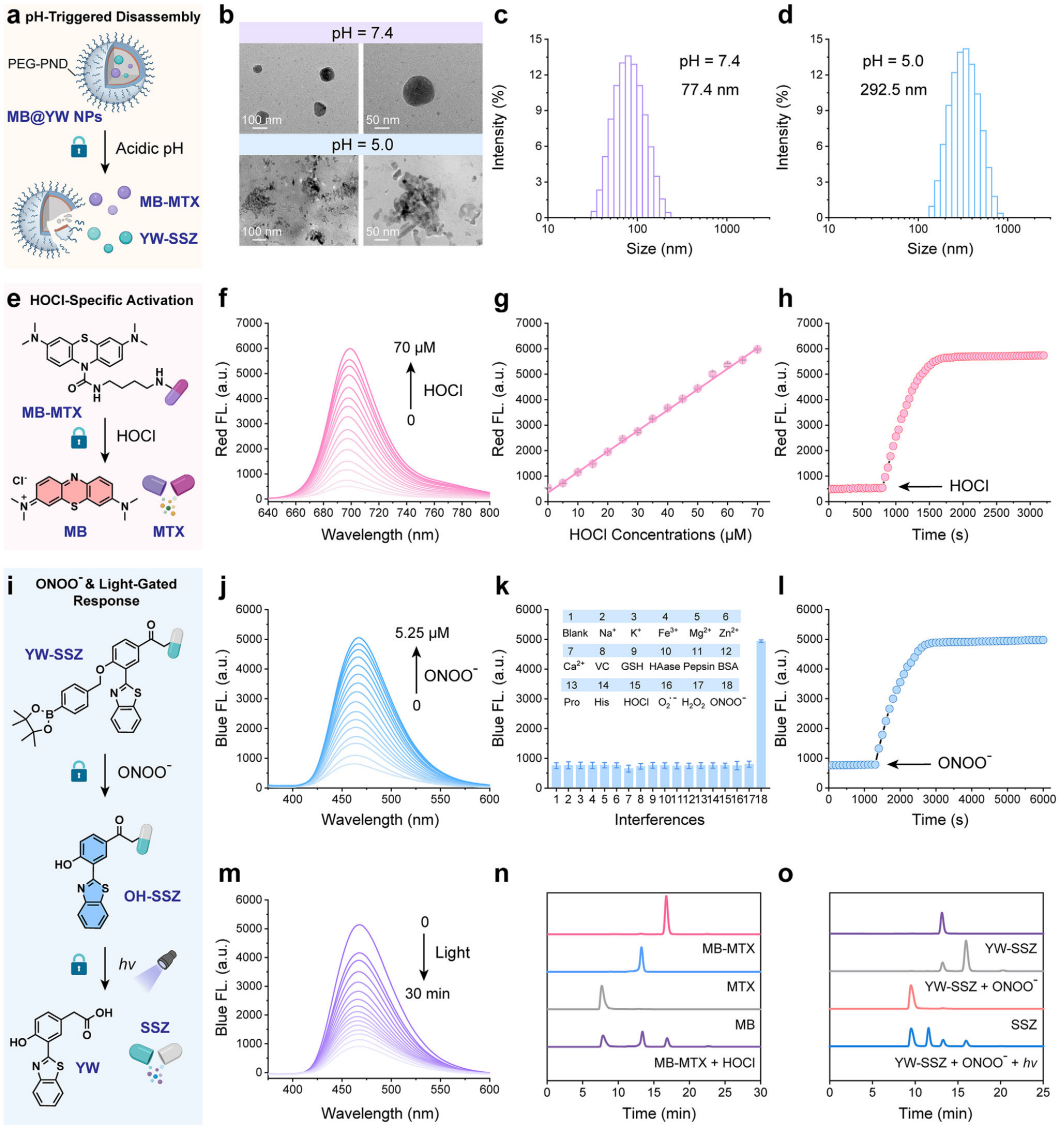
Physicochemical characterization and hierarchical pH/oxidant/light‐responsive behavior of MB@YW NPs. (a) Schematic illustration of the acidic pH‐triggered disassembly of MB@YW NPs (first lock activation). (b) Representative TEM images and (c,d) corresponding hydrodynamic size distributions of MB@YW NPs incubated at pH 7.4 and 5.0. (e) Schematic representation of the HOCl‐mediated activation of the MB‐MTX prodrug (second lock activation). (f) Fluorescence spectra of MB@YW NPs upon the addition of HOCl (70 µM) under acidic conditions (pH 5.0). (g) Linear correlation between the NIR fluorescence intensity at 699 nm and HOCl concentration. (h) Photostability evaluation of the HOCl‐activated MB@YW NPs. (i) Schematic illustration of the sequential ONOO^−^‐ and light‐triggered (400–650 nm) activation of the YW‐SSZ prodrug (second‐to‐third lock cascade process). (j) Fluorescence spectra of MB@YW NPs treated with ONOO^−^ (5.25 µM) under acidic conditions (pH 5.0). (k) Selectivity profile of the MB@YW NPs toward ONOO^−^ over various biologically relevant interferents (blue channel). (l) Photostability of the ONOO^−^‐activated MB@YW NPs. (m) Time‐dependent fluorescence attenuation of ONOO^−^‐treated MB@YW NPs upon light irradiation (80 mW cm^−2^, 400–650 nm). (n,o) HPLC chromatograms validating the specific cleavage and drugs liberation from (n) MB‐MTX by HOCl and (o) YW‐SSZ by sequential ONOO^−^ and light treatments. Concordant results were obtained from three independent experiments.

This hierarchical integration of protonation, oxidation, and photolysis establishes an orthogonally branched, triple‐locked logic‐gated architecture for spatiotemporal signal processing, enabling real‐time, self‐monitored, and orthogonally programmable drug delivery. The molecular precision of these synthetic components was rigorously validated via high‐resolution mass spectrometry and ^1^H/^13^C NMR spectroscopy, providing a robust chemical foundation for this spatiotemporally coordinated and programmable intervention strategy.

### 2.2 Triple‐Locked Disassembly and Orthogonal Drug Release

Upon exposure to physiological and pathological pH environments, we first evaluated the pH‐responsive “first lock” governing structural disassembly of MB@YW NPs. At physiological pH 7.4, the nanoplatform maintained a stable, monodisperse spherical morphology (∼77.4 nm, PDI 0.178) with negligible size variation over 66 h, confirming excellent colloidal stability for systemic circulation (Figure 1a–c and Figure S1). Crucially, to verify its responsiveness in the physiologically relevant mildly acidic microenvironment of RA synovial fluid, the nanoparticles were exposed to pH 6.5. Upon incubation at pH 6.5, protonation of the PEG‐PND shell triggered structural loosening and disassembly, accompanied by an increase in hydrodynamic size (∼177.9 nm) and a distinct surface charge reversal from –1.31 to +0.68 mV (Figure S1). When the acidity was further increased to pH 5.0, this protonation effect became more pronounced, leading to complete structural collapse into irregular aggregates (∼292.5 nm) and a further elevated positive surface charge (+1.51 mV) (Figure 1b,d and Figure S1). These progressive structural and electrical transitions demonstrate that MB@YW NPs possess sensitive pH responsiveness, enabling efficient disassembly and cargo release across the acidic microenvironmental gradient of the inflamed joints.

We subsequently investigated the intracellular “second lock” driven by oxidative stress, which governs the chemically orthogonal activation of the two prodrugs. Focusing initially on the HOCl‐responsive MB‐MTX axis, we observed that exposure to escalating HOCl concentrations under acidic conditions provoked a robust, concentration‐dependent 11.2‐fold enhancement in NIR fluorescence at 699 nm, a response directly attributed to the selective oxidative cleavage of the urea linkage (Figure 1e,f). Quantitative analysis revealed an exceptionally strong linear correlation (*F*_699 nm_ = 81.13[HOCl] + 345.45, R^2^ = 0.9985) between the NIR signal and HOCl concentration, together with high selectivity against other ROS, metal ions, and reducing species under acidic conditions (pH 5.0; Figure 1g and Figure S2). Importantly, to better mimic the pathological arthritic microenvironment, the selectivity was further confirmed under mildly acidic conditions (pH 6.5), where MB@YW NPs consistently displayed minimal cross‐reactivity toward biologically relevant interferents (Figure S3), confirming robust and reliable oxidative unlocking across relevant pathological pH ranges. Moreover, the fluorescence signal exhibited excellent photostability over extended durations, a critical attribute for continuous in vivo detection (Figure 1h). Crucially, the isolated small‐molecule prodrug MB‐MTX exhibited an identical HOCl‐responsive profile (Figure S4), confirming that this sensitive oxidative unlocking is intrinsically rooted in its molecular design and successfully preserved within the nanoplatform.

In parallel, the sensitivity of the MB@YW nanoplatform toward ONOO^−^ was interrogated under acidic conditions (Figure 1i). Upon the addition of ONOO^−^, the disassembled system exhibited a robust, concentration‐dependent surge in blue fluorescence at 468 nm, achieving a 6.8‐fold enhancement (Figure 1j and Figure S5). This response demonstrated exceptional specificity, remaining effectively locked in the presence of various competing ROS and biological interferents (Figure 1k). The signaling remained stable over 1 h, confirming the platform's reliability for sustained monitoring (Figure 1l).

Having validated the ONOO^−^‐triggered fluorescence activation, the terminal photo‐triggered “third‐lock” for SSZ liberation was subsequently explored. As shown in Figure 1m, irradiation (400–650 nm) of the ONOO^−^‐activated system induced a progressive attenuation of the blue fluorescence, providing a real‐time optical readout of the drug release process. Mechanistically, this is driven by an ESIPT process followed by a photo‐Favorskii rearrangement within the YW‐SSZ moiety, which triggers the liberation of the SSZ drug cargo and yields YW as the terminal product (Figure S6). Notably, the concomitant decline in blue fluorescence directly correlates with SSZ drug release, enabling the MB@YW NPs to function as a self‐reporting delivery vehicle for offering high‐fidelity spatiotemporal control over drug activation in RA. Mirroring the HOCl responsive behavior, the free YW‐SSZ molecules exhibited an identical fluorescence signature characterized by ONOO^−^‐mediated activation and light‐triggered quenching (Figures S7 and S8).

To directly verify the stimuli‐responsive drug release, the stepwise cleavage of both prodrugs was monitored by high‐performance liquid chromatography (HPLC). Upon HOCl stimulation, the MB‐MTX prodrug peak (*R*_T_ = 16.75 min) rapidly diminished, accompanied by the emergence of distinct peaks for the free MB fluorophore (*R*_T_ = 7.68 min) and MTX cargo (*R*_T_ = 13.26 min), confirming efficient urea bond cleavage (Figure 1n). Correspondingly, the stepwise activation of YW‐SSZ was chromatographically mapped (Figure 1o). Exposure to ONOO^−^ induced the consumption of YW‐SSZ (*R*_T_ = 13.15 min) and the appearance of a new intermediate peak at *R*_T_ = 15.95 min, consistent with ONOO^−^‐mediated removal of the pinacol boronate moiety, with negligible premature SSZ release (Figure 1o). Upon subsequent photo‐irradiation (400–650 nm), this intermediate was further converted, leading to the release of authentic SSZ, as verified by comparison with an authentic SSZ standard at *R*_T_ = 9.50 min.

These results confirm that MB@YW NPs operate through a hierarchically gated three‐lock system, integrating pH‐triggered structural disassembly, chemically orthogonal HOCl/ONOO^−^ activation, and light‐controlled terminal release. This design enables fully decoupled spatiotemporal regulation of dual prodrugs, supported by consistent fluorescence evolution and chromatographic validation across all stages.

### 2.3 Intracellular Orthogonal Decoding and Spatiotemporally Cascaded Synergistic Therapy

Inflamed macrophages in RA feature oxidative bursts (HOCl, ONOO^−^) and metabolic dysregulation, requiring a nanoplatform that can orthogonally decode these cues for coordinated drug release. MB@YW NPs fulfill this via HOCl‑triggered MTX release (NIR turn‑on) and ONOO^−^/light‑gated SSZ release (blue on‑to‑off), enabling real‑time dual‑channel monitoring and cascaded synergistic therapy. We next examined their intracellular performance in signal decoding, ROS scavenging, ATP restoration, and anti‑inflammatory efficacy (Figure 2a).

**FIGURE 2:**
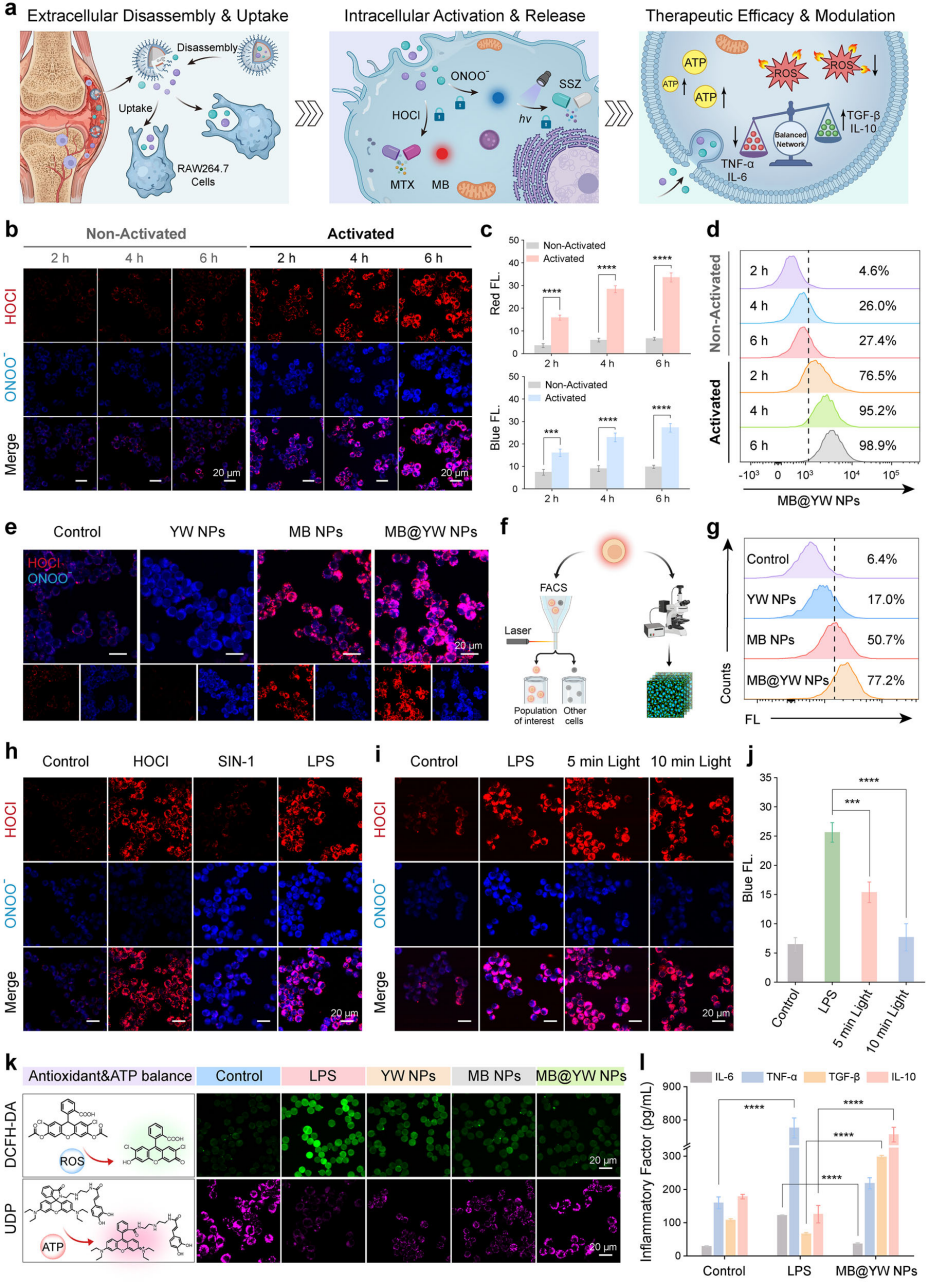
Intracellular orthogonal decoding and spatiotemporally cascaded synergistic therapy of MB@YW NPs. (a) Schematic illustration showing the extracellular disassembly, intracellular activation, and multifaceted therapeutic mechanisms of MB@YW NPs. (b) Representative CLSM images of quiescent (non‐activated) and LPS‐activated RAW264.7 cells after incubation with MB@YW NPs for 2, 4, and 6 h. (c) Quantitative analysis of dual‐channel fluorescence intensities corresponding to the images in (b). (d) Flow cytometric evaluation of MB@YW NP internalization based on the red fluorescence channel by quiescent and LPS‐activated RAW264.7 cells across different incubation periods (2, 4, and 6 h). (e) CLSM images of LPS‐activated RAW264.7 cells following 6 h incubation with various nanoparticle formulations. (f) Schematic representation of the intracellular orthogonal sensing mechanism by CLSM and flow cytometry. (g) Comparative flow cytometric quantification of nanoparticle uptake (MB NPs, YW NPs, and MB@YW NPs) in LPS‐activated RAW264.7 cells. (h) Selective fluorescence activation in red and blue channels within RAW264.7 cells treated with MB@YW NPs (6 h) upon challenge with exogenous (HOCl, SIN‐1) or endogenous (LPS‐induced) stimuli. (i) CLSM images demonstrating the drugs release in control and LPS‐activated RAW264.7 cells before and after photo‐irradiation. (j) Corresponding quantitative analysis of blue fluorescence attenuation from the images in (i). (k) Representative fluorescence imaging of intracellular ROS levels (DCFH‐DA) and ATP‐associated signals (UDP) in LPS‐activated RAW264.7 cells following various treatments. (l) Secretion levels of pro‐inflammatory (TNF‐α, IL‐6) and anti‐inflammatory (TGF‐β, IL‐10) cytokines in control, LPS‐activated, and MB@YW NP‐treated RAW264.7 cells, as determined by ELISA. The data are expressed as the mean ± SD. Statistical significance was determined using Student's t‐test for pairwise comparisons, and a one‐way ANOVA analysis of variance for multiple groups. ^***^ *p* < 0.001, ^****^ *p* < 0.0001. Concordant results were obtained from three independent experiments.

Herein, we first assessed the biocompatibility and inflammatory targeting behavior of MB@YW NPs in macrophages. Hemolysis assays demonstrated negligible erythrocyte disruption even at high concentrations (240 µg/mL), with hemoglobin release comparable to the negative control (Figure S9). In parallel, CCK‐8 assays confirmed high cell viability (>85%) in RAW264.7 macrophages, indicating excellent cytocompatibility. Furthermore, to rule out potential phototoxicity or photothermal artifacts during light‐activated release, both cell viability and solution temperature under continuous light irradiation (400‐650 nm) were monitored. CCK‐8 assays verified that the applied irradiation caused negligible phototoxicity to macrophages (Figure S10). Concurrently, real‐time infrared thermal imaging revealed virtually unchanged temperatures in both nanoparticle solutions and buffer controls (Figure S11), firmly excluding any hyperthermia‐related contributions. To evaluate inflammation‐selective uptake, LPS‐stimulated macrophages were used to mimic the RA inflammatory microenvironment. Confocal laser scanning microscopy (CLSM) revealed markedly enhanced intracellular fluorescence in activated macrophages, whereas negligible signals were observed in resting cells (Figure 2b,c). Flow cytometry further confirmed this selective internalization, with 98.9% fluorescence‐positive cells at 6 h and a 4.4‐fold increase in mean fluorescence intensity (MFI) in the activated group compared to controls (Figure 2d and Figure S12). These results establish efficient inflammation‐guided cellular uptake as the prerequisite for intracellular decoding.

We next investigated intracellular orthogonal decoding behavior. In LPS‐stimulated cells, MB@YW NPs generated simultaneous red and blue fluorescence outputs, whereas MB NPs and YW NPs produced only single‐channel responses, confirming decoupled signal generation (Figure 2e,f and Figure S13). Flow cytometry analysis further demonstrated enhanced red‐channel intensity in MB@YW‐treated cells compared with single‐prodrug controls (Figure 2g and Figure S12). To further confirm chemical orthogonality, cells were challenged with specific stimuli. Exogenous HOCl selectively enhanced the red fluorescence channel, while ONOO^−^ triggered specific activation of the blue channel (Figure 2h). In contrast, LPS‐induced endogenous oxidative stress activated both channels simultaneously (Figure S14). Collectively, these results demonstrate that MB@YW NPs function as an intracellular orthogonal decoding system capable of independently resolving HOCl‐ and ONOO^−^‐associated inflammatory signals.

We further demonstrated that intracellular decoding is tightly coupled with therapeutic activation. In LPS‐activated macrophages, HOCl triggered MTX release from MB‐MTX, accompanied by a pronounced enhancement of the red fluorescence signal (Figure 2i and Figure S15). Subsequently, light irradiation induced time‐dependent attenuation of the blue fluorescence channel, while the red signal remained stable, indicating selective SSZ release via the third lock (Figure 2i,j and Figure S15). These results confirm a temporally ordered cascade of MTX release followed by SSZ activation, with dual‐channel fluorescence serving as a real‐time reporter of drug release dynamics.

Finally, functional assays showed that MB@YW NPs significantly reduced intracellular ROS levels, restored ATP‐associated bioenergetic signals, and reprogrammed cytokine secretion toward an anti‐inflammatory phenotype, outperforming single‐drug controls (Figure 2k,l and Figures S16 and S17) [46, 47]. Collectively, these results demonstrate that MB@YW NPs enable intracellular orthogonal decoding of inflammatory signals and translate them into spatiotemporally cascaded therapeutic responses. This integrated design bridges microenvironment sensing and programmable drug activation, providing a generalizable strategy for precision reprogramming of dysfunctional immune cells in RA.

### 2.4 Spatiotemporal In Vivo Tracking and Targeted Accumulation in Arthritic Lesions

MB@YW NPs are designed to remain optically silent during systemic circulation and undergo pH‐triggered activation specifically in the acidic synovium. To evaluate whether this cascaded activation strategy can be faithfully executed in vivo, we first investigated their real‐time biodistribution and fluorescence dynamics in a λ‐carrageenan‐induced rat arthritis model (Figure 3a) [48, 49]. SD rats were categorized into bilateral arthritis, unilateral arthritis, and healthy control groups. Following intravenous administration, whole‐body fluorescence imaging revealed a rapid and pronounced signal emergence specifically at inflamed joints, whereas healthy animals exhibited negligible fluorescence throughout the observation period (Figure 3b,c). Quantitative analysis of the left hind paw showed a 4.2‐fold increase in fluorescence intensity in the bilateral arthritis group at 6 h post‐injection compared with that at 0.5 h, and the signal was 11.8‐fold higher than that in healthy controls (Figure 3c–e). In the unilateral arthritis model, fluorescence was strictly confined to the affected limb, confirming precise pathology‐gated activation with minimal off‐target signal (Figure 3d). These results collectively indicate that MB@YW NPs remain optically silent during systemic circulation and are selectively activated only within diseased joints.

**FIGURE 3:**
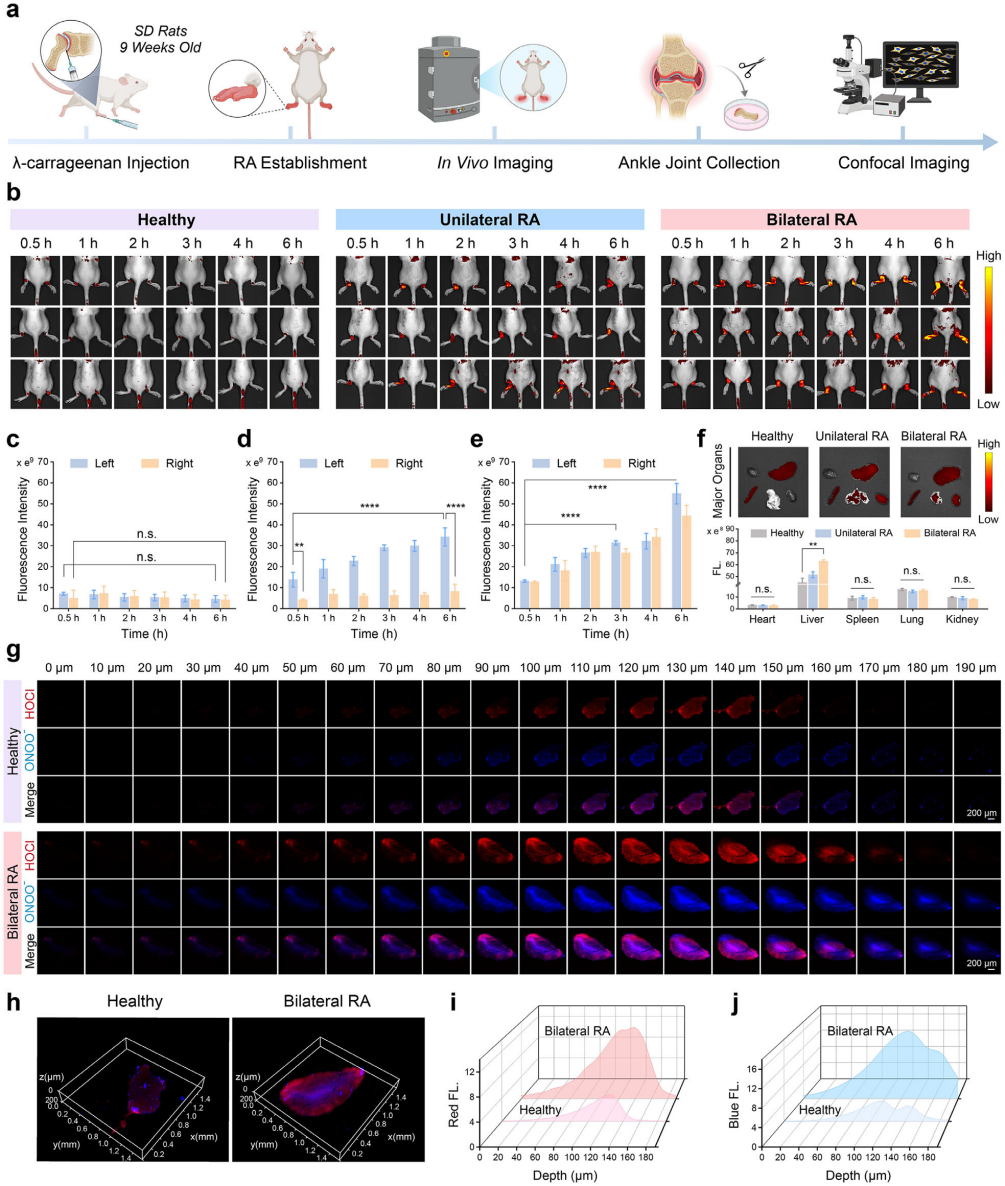
Spatiotemporal in vivo tracking and deep penetration of MB@YW NPs. (a) Schematic illustration of the experimental timeline for the *λ*‐carrageenan‐induced SD rat arthritis model establishment and subsequent in vivo fluorescence imaging. (b) Representative time‐course in vivo fluorescence images of healthy, unilateral RA, and bilateral RA rats at predetermined time points (0.5–6 h) following intravenous administration of MB@YW NPs (fluorescence signal collected using an IVIS imaging system with λ_ex_ = 620 nm, λ_em_ = 710 nm). (c‐e) Corresponding quantitative analysis of the time‐dependent fluorescence intensities in the left and right hind paws of the respective groups. (f) Ex vivo fluorescence images and quantitative biodistribution in major organs (heart, liver, spleen, lung, and kidneys) harvested at 6 h post‐injection. (g) Representative *ex vivo* confocal z‐stack images of excised ankle joints from healthy and bilateral RA rats at 6 h post‐injection. (h) Three‐dimensional (3D) reconstructed confocal images of ankle joints from healthy and bilateral RA rats. (i,j) Quantitative depth‐dependent profiles of red and blue fluorescence intensities in the ankle joint tissues of healthy and bilateral RA rats. The data are expressed as the mean ± SD. Statistical significance in (c–e) was determined by repeated‐measures two‐way ANOVA followed by Sidak's (for paw comparisons) or Tukey's (for time‐course comparisons) post hoc multiple‐comparison test. Statistical significance in (f) was analyzed by one‐way ANOVA followed by Tukey's post hoc test. ^**^ *p* < 0.01, ^***^ *p* < 0.001, ^****^ *p* < 0.0001. Three rats in each group (*n* = 3).

To further assess intratissue distribution, ex vivo imaging and z‐stack confocal analysis were performed on harvested ankle joints. *Ex vivo* fluorescence imaging confirmed strong signal retention in arthritic tissues (Figure 3g), while negligible fluorescence was observed in major organs except for liver accumulation, suggesting hepatic clearance as the primary metabolic pathway (Figure 3f). Three‐dimensional confocal reconstruction revealed that fluorescence signals in arthritic joints penetrated deeply into synovial tissues, extending from 0 to 190 µm with strong and homogeneous distribution (Figure 3g–j). In contrast, healthy joints exhibited only weak and superficial peripheral signals, indicating limited probe penetration under non‐inflammatory conditions. These findings confirm that MB@YW NPs achieve not only lesion‐specific accumulation but also deep tissue infiltration within inflamed synovial microenvironments.

The observed in vivo spatiotemporal specificity arises from the hierarchical activation logic of the platform. Specifically, the acidic synovial microenvironment triggers first‐lock disassembly of the PEG‐PND shell, enabling enhanced joint retention and cellular uptake, followed by intracellular oxidative stress‐driven second‐lock activation that induces localized fluorescence signaling and drug release within arthritic lesions. This cascaded activation ensures lesion‐restricted signal generation, enabling high‐contrast visualization and precise inflammatory mapping in vivo.

### 2.5 Real‐Time Theranostic Feedback and Synergistic Reprogramming of Arthritic Microenvironment

MB@YW NPs are designed to provide real‐time dual‐channel fluorescence readouts (NIR turn‐on for MTX release and blue on‐to‐off for SSZ release) upon sequential pH/oxidant/light activation, enabling non‐invasive monitoring of therapeutic delivery and efficacy. To evaluate the in vivo translational potential of the programmable MB@YW NPs, we first established a highly clinically relevant collagen‐induced arthritis (CIA) model in DBA/1 mice. Briefly, the mice received a primary immunization via a subcutaneous injection of bovine type II collagen emulsion [27, 50]. To robustly break immune tolerance and trigger systemic inflammation, a booster immunization was administered on day 21. Following this cascaded induction, successful disease onset was confirmed by day 28, which was morphologically characterized by progressive paw swelling and steadily elevated clinical scores. With the severe arthritic phenotype firmly established, the mice were randomized into different cohorts and subjected to a 15‐day targeted treatment regimen starting on day 30. Specifically, the mice were intravenously administered either PBS (untreated RA), YW NPs, MB NPs, or MB@YW NPs coupled with localized irradiation every three days (Figure 4a).

**FIGURE 4:**
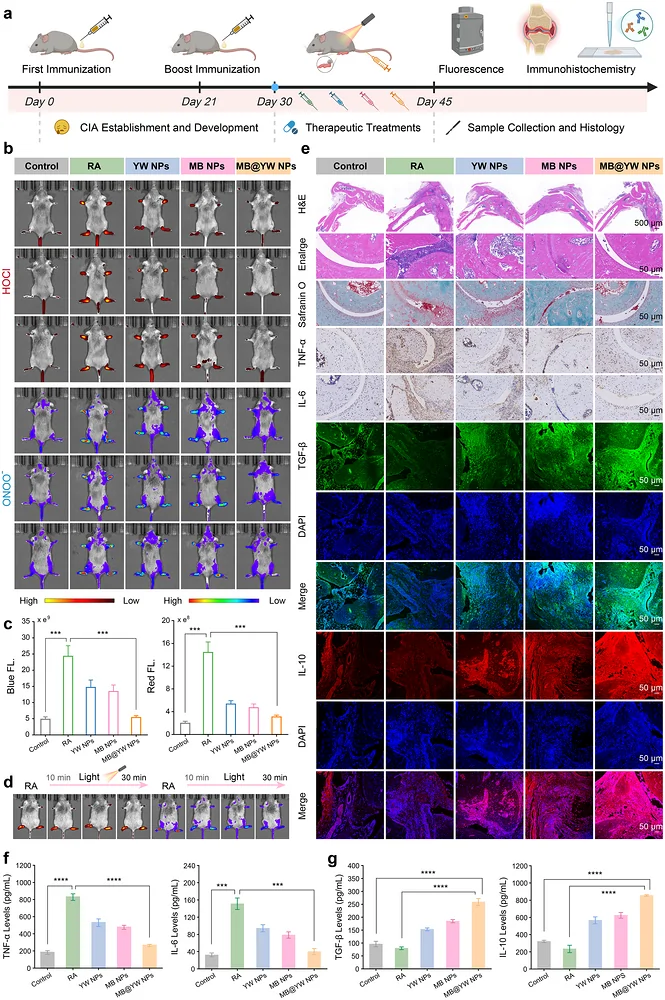
Real‐time theranostic feedback and synergistic reprogramming of the arthritic microenvironment. (a) Schematic illustration of the experimental timeline, detailing CIA model establishment, therapeutic treatments and sample collection and histology. (b) Representative in vivo fluorescence images assessing therapeutic efficacy of MB@YW NPs (Red channel: λ_ex_ = 620 nm, λ_em_ = 710 nm; Blue channel: λ_ex_ = 420 nm, λ_em_ = 520 nm). (c) Quantification of blue and red fluorescence signals from the joint regions in (b). (d) Real‐time, dual‐channel self‐monitoring of the delivery process of MB@YW NPs. (e) Histological analysis and immunohistochemical staining of ankle sections. (f,g) ELISA‐based quantification of cytokine concentrations (TNF‐α, IL‐6, TGF‐β, and IL‐10) in joint tissue from all treatment groups. The data are expressed as the mean ± SD. Statistical significance was determined using Student's t‐test for pairwise comparisons, and a one‐way ANOVA analysis of variance for multiple groups. ^***^ *p* < 0.001, ^****^ *p* < 0.0001. Three mice in each group (*n* = 3).

To assess theranostic feedback capacity, we explored the potential of our nanoplatform for monitoring treatment outcomes via activatable dual‐channel fluorescence imaging. At the end of the therapeutic window, all cohorts received a final diagnostic intravenous injection of MB@YW NPs, and their in vivo biodistribution and joint inflammatory status were dynamically monitored 6 h post‐injection. As shown in Figure 4b, the untreated RA group exhibited intense fluorescence signals in the ankle joints, attributed to excessive ROS and RNS accumulation and the highly acidic microenvironment. Notably, the blue and red fluorescence intensities in mice treated with MB@YW NPs decreased to merely 22.1% and 21.3% of those in the untreated RA group, respectively, providing direct, non‐invasive visual evidence for the profound alleviation of joint inflammation (Figure 4c).

To elucidate the spatiotemporal drug liberation dynamics, we further performed real‐time, dual‐channel self‐monitoring of the delivery process. Upon intravenous injection of MB@YW NPs into CIA mice followed by continuous localized irradiation, real‐time imaging revealed a robust emergence of red fluorescence in the inflamed ankles, corroborating the successful targeted delivery of MTX (Figure 4d). Concurrently, a progressive attenuation of the initially activated blue fluorescence was observed upon extended light exposure, accurately reporting the successful, light‐gated liberation of SSZ (Figure S18). These imaging readouts collectively demonstrate that MB@YW NPs enable a highly reliable, dual‐channel self‐monitoring mechanism, providing real‐time feedback for non‐invasive evaluation of both drug release and therapeutic efficacy.

Corroborating these in vivo imaging readouts, comprehensive histopathological evaluations were performed on the harvested ankle joints (Figure 4e). Hematoxylin and eosin (H&E) staining of the untreated RA joints revealed rampant immune cell infiltration, severe bone erosion, and marked synovial hyperplasia, indicative of aggressive and uncontrolled polyarthritis. Treatment with YW NPs or MB NPs alone merely afforded partial mitigation, while inflammatory infiltration was modestly reduced, substantial cartilage damage and structural deformities persisted. In striking contrast, joints treated with MB@YW NPs exhibited a near‐complete restoration toward normal architecture. Only negligible leukocyte infiltration was observed, and the articular surfaces displayed well‐preserved, clearly delineated bone margins. To rigorously assess cartilage integrity, Safranin O–Fast Green staining was performed (Figure 4e). While the RA group suffered from extensive cartilage degradation and severely deformed bone‐cartilage interfaces, MB@YW NPs treatment robustly preserved a continuous, smooth cartilage layer, effectively shielding the joints from progressive inflammatory destruction.

Since RA is fundamentally driven by a dysregulated immune microenvironment, we sought to dissect the metabolic and immunomodulatory mechanisms underlying this profound structural protection. We first evaluated the biochemical reprogramming of the arthritic joints (Figure S19). While untreated RA tissues exhibited severe oxidative stress and bioenergetic depletion, MB@YW NPs robustly scavenged excessive ROS and restored local ATP to near‐physiological levels. Building upon this critical metabolic rescue, we further investigated the subsequent immunomodulation. Immunohistochemical (IHC) analysis was conducted to evaluate the local expression of pro‐inflammatory cytokines (IL‐6 and TNF‐α), which are central drivers of RA pathogenesis. Ankle sections from untreated RA mice exhibited intensely elevated IL‐6 and TNF‐α levels (Figure 4e). While monotherapies induced a noticeable but incomplete reduction, MB@YW NPs elicited the most potent inhibitory effect, suppressing these pro‐inflammatory markers to near‐physiological levels. Furthermore, we investigated whether MB@YW NPs actively instigate an inflammation‐resolving milieu by assessing anti‐inflammatory cytokines (TGF‐β and IL‐10). Strikingly, the MB@YW NPs cohort displayed the most robust TGF‐β and IL‐10 signals among all groups, signifying a potent reactivation of anti‐inflammatory pathways (Figure 4e).

To quantitatively substantiate this coordinated immunomodulation, cytokine levels in joint tissue homogenates were measured via ELISA (Figure 4f,g). Consistent with histological findings, IL‐6 and TNF‐α concentrations in MB@YW NPs‐treated mice were drastically curtailed to 26.5% and 31.9% of those in the RA group, respectively (Figure 4f and Figure S20). Conversely, TGF‐β and IL‐10 levels surged by 3.3‐fold and 3.7‐fold compared to the untreated cohort, with monotherapies producing only intermediate effects (Figure 4g and Figure S20). Collectively, these results unambiguously demonstrate that MB@YW NPs fundamentally rewire the arthritic microenvironment by synergistically coupling metabolic rescue with profound immune reprogramming to halt RA progression.

### 2.6 Synergistic In Vivo Amelioration of Arthritis and Comprehensive Bone Preservation

Encouraged by this profound immune reprogramming and real‐time diagnostic feedback, we next systematically evaluated how this microenvironmental modulation translates into macroscopic clinical benefits and comprehensive bone preservation (Figure 5a). By the end of the therapeutic window, macroscopic evaluation revealed stark phenotypic contrasts. Untreated RA mice exhibited severe polyarthritis with pervasive edema, erythema, and clinical scores peaking at 12 (Figure 5b,c). In striking contrast, treatment with MB@YW NPs nearly abrogated these macroscopic symptoms, maintaining paw morphologies akin to healthy controls (Figure 5b,c). This robust clinical amelioration underscores the in vivo efficacy of our triple‐locked delivery, orthogonal prodrug activation strategy in quenching localized inflammation.

**FIGURE 5:**
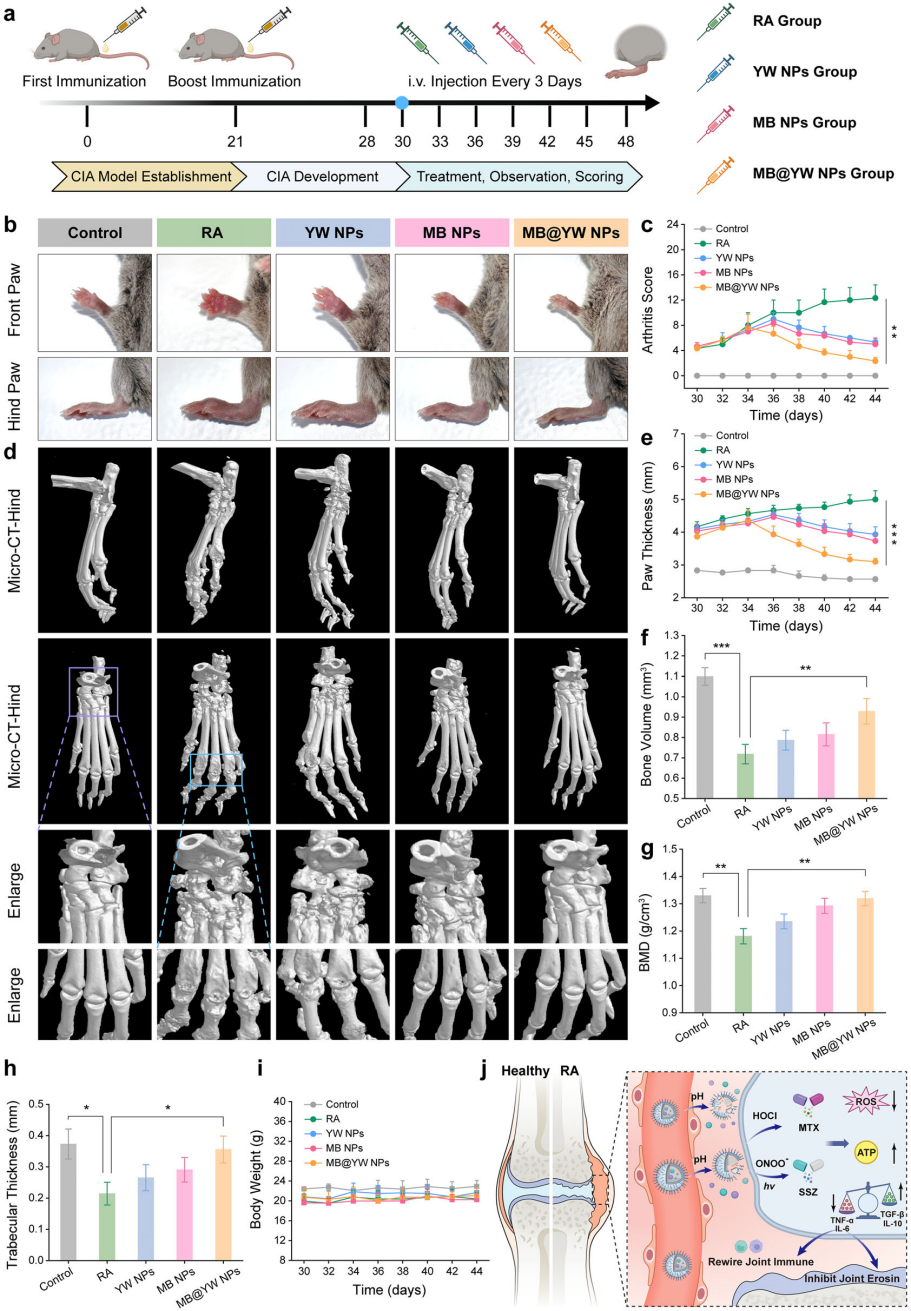
Synergistic in vivo amelioration of arthritis and comprehensive bone preservation by MB@YW NPs. (a) Schematic illustration of the experimental timeline, detailing CIA model establishment, CIA development and therapeutic regimen. (b,d) Representative macroscopic photographs (b) and 3D micro‐CT reconstructions (d) of the front and hind paws from different treatment cohorts at the study endpoint. (c,e–i) Quantitative evaluations of therapeutic outcomes, including clinical arthritis scores (c), paw thickness (e), bone volume (f), bone mineral density (g), trabecular thickness (h), and body weight changes (i). (j) Schematic illustration of the therapeutic mechanisms of MB@YW NPs within the inflamed joints, highlighting the rewiring of the local immune microenvironment and the inhibition of bone erosion. The data are expressed as the mean ± SD. Statistical significance was determined using Student's t‐test for pairwise comparisons, and a one‐way ANOVA analysis of variance for multiple groups. ^*^ *p* < 0.05, ^**^ *p* < 0.01, ^***^ *p* < 0.001. Three mice in each group (*n* = 3).

To rigorously quantify the preservation of bone microarchitecture, which is a critical metric for evaluating RA interventions, high‐resolution micro‐computed tomography (micro‐CT) was performed on the excised ankle joints. As shown in Figure 5d, the untreated RA mice displayed extensive bone destruction, characterized by irregular and eroded joint surfaces, marked cartilage degeneration, reduced bone volume, and pronounced decreases in trabecular thickness and bone mineral density (BMD). Monotherapies utilizing either YW NPs or MB NPs yielded merely modest therapeutic benefits, leaving considerable structural damage unresolved. Remarkably, MB@YW NPs treatment led to a substantial restoration of bone structural integrity, manifested by clearly delineated bone contours, relatively smooth articular surfaces, and a visibly more preserved joint architecture (Figure 5d). Quantitative morphometric analysis unambiguously validated these observations: MB@YW NPs significantly enhanced bone volume (by ∼29.2%, Figure 5f) while comprehensively restoring BMD (Figure 5g) and trabecular thickness (Figure 5h) to near‐normal levels. These data collectively indicate that MB@YW NPs effectively inhibit RA‐driven bone loss and cartilage erosion, highlighting a synergistic therapeutic effect that surpasses the efficacy of the respective monotherapies.

Longitudinal monitoring further substantiated the sustained therapeutic dominance of the nanoplatform. Throughout the regimen, MB@YW NPs decisively suppressed the escalation of arthritis scores and paw thickness (Figure 5c,e), reflecting a continuous and dynamic reprogramming of the inflammatory milieu. Crucially, the systemic safety of this programmable platform was validated by stable body weights and the absence of histological damage in major organs across all treatment groups (Figure 5i and Figure S21). Comprehensive biochemical analyses confirmed that liver function markers, including alanine aminotransferase (ALT), aspartate aminotransferase (AST), and albumin, as well as renal indicators such as creatinine and urea, remained well within physiological ranges (Figure S22). Furthermore, hematological parameters including white blood cell (WBC) counts, hemoglobin (HGB) levels, and platelet (PLT) indices showed no significant deviations from healthy controls (Figure S23), underscoring the favorable biocompatibility and low systemic safety profile of the MB@YW NPs. Taken together, these results unambiguously demonstrate that MB@YW NPs successfully translate microenvironmental modulation into profound macroscopic clinical remission and comprehensive structural preservation (Figure 5j). By synergistically halting bone erosion while maintaining exceptional systemic biosafety, this programmable nanoplatform establishes a highly translatable and paradigm‐shifting strategy for RA theranostics.

### 2.7 Transcriptomic Reprogramming of RA Inflammation via Rewiring cAMP/NF‐κB Signaling Axis

To elucidate the molecular mechanisms underlying the profound therapeutic efficacy of MB@YW NPs, we performed RNA‐sequencing (RNA‐seq) transcriptomic profiling on RAW264.7 macrophages across healthy controls, the in vitro RA model, and the MB@YW NPs‐treated group. The Venn diagram analysis identified a total of 2523 differentially expressed genes (DEGs) in the RA group relative to the healthy control, whereas 434 genes were significantly altered following MB@YW intervention, indicating a targeted transcriptomic modulation (Figure S24). Among these, 294 genes (67.7%) directly overlapped with the RA‐associated DEGs, with 290 of them showing no significant difference between the MB@YW NPs and healthy control groups, indicating a targeted normalization of disease‐perturbed genes. The volcano plots further revealed that the induction of the RA phenotype caused a drastic perturbation of the transcriptomic landscape, resulting in 1713 significantly upregulated and 810 downregulated genes (Figure 6a). Remarkably, this broad inflammatory transcriptional signature was robustly reversed following intervention with MB@YW NPs, which effectively downregulated 262 genes and upregulated 172 genes compared with the untreated RA group (Figure 6b). Hierarchical clustering of global gene expression profiles further visualized this therapeutic reversal, confirming an opposite regulatory trend: the massive cluster of genes strongly upregulated upon RA modeling was substantially suppressed after MB@YW NPs intervention, yielding a global transcriptomic pattern highly analogous to that of the healthy control state (Figure 6d).

**FIGURE 6:**
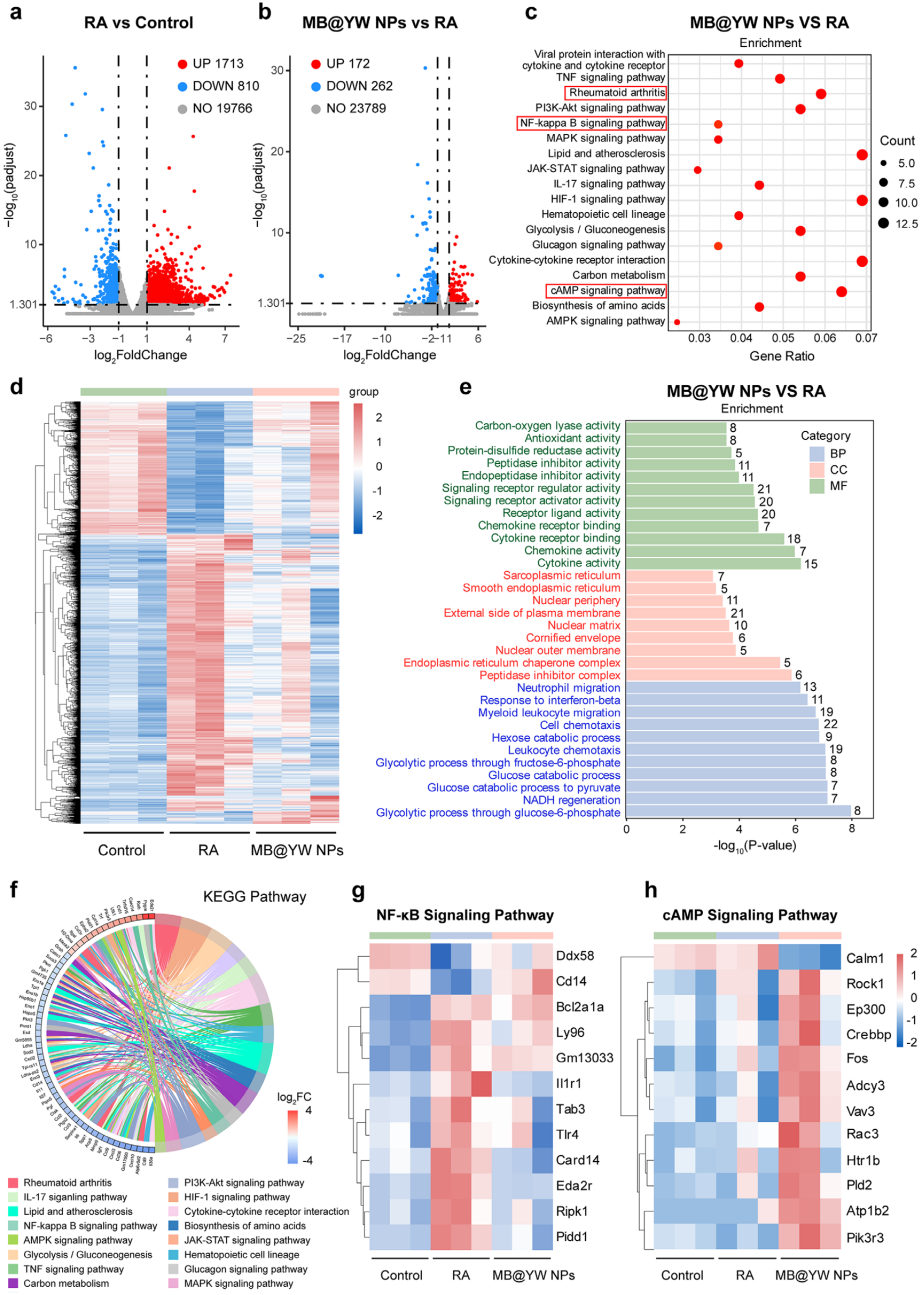
Transcriptomic profiling of RAW264.7 cells following MB@YW NPs treatment. (a) Volcano plot of differentially expressed genes between RA model and control groups. (b) Volcano plot of differentially expressed genes between MB@YW NPs‐treated RA model and RA model groups. (c) KEGG pathway enrichment analysis of inflammation‐ and immunity‐related differentially expressed genes (DEGs) between RA model and MB@YW NPs‐treated RA model RAW264.7 cells. (d) Heat map of global gene expression profiles in control RAW264.7 cells (green), RA model RAW264.7 cells (blue), and MB@YW NPs‐treated RA model RAW 264.7 cells (red). Upregulated genes are shown in red and downregulated genes in blue. The color bar denotes Z‐scores of normalized expression values. (e) GO enrichment analysis of DEGs between RA model and MB@YW NPs‐treated RA model RAW264.7 cells. (f) Chord diagram illustrating enriched KEGG pathways between RA model and MB@YW NPs‐treated RA model RAW264.7 cells. (g,h) Heat maps showing DEGs in the NF‐κB signaling pathway (g), and cAMP signaling pathway (h), respectively.

To define the biological networks remodeled by MB@YW NPs, we performed pathway‐level analyses of the DEGs identified between the MB@YW NPs‐treated and untreated RA groups. Kyoto Encyclopedia of Genes and Genomes (KEGG) enrichment analysis revealed that these DEGs were mainly associated with immune and inflammatory pathways closely linked to RA progression, including the rheumatoid arthritis pathway, TNF signaling, IL‐17 signaling, cytokine‐cytokine receptor interaction, and NF‐κB signaling, a central mediator of inflammatory cytokine production (Figures 6c, f) [51, 52, 53]. Notably, the cAMP signaling pathway was also significantly enriched. Expression heatmaps across these enriched cascades further revealed that hallmark disease‐induced inflammatory genes (such as *Il6*, *Il1b*, *Il18*, and *Ccl2* in the RA pathway) were markedly suppressed following MB@YW NPs treatment (Figure S25).

Although multiple inflammatory cascades were concurrently identified, we prioritized the cAMP and NF‐κB signaling axis as the primary regulatory hub based on both the pharmacological actions of the encapsulated payloads and the signaling hierarchy in macrophages. Pharmacologically, the two active DMARD payloads co‐delivered by MB@YW NPs, sulfasalazine (SSZ) and methotrexate (MTX), directly target this regulatory axis: SSZ functions as an established inhibitor of IκB kinases that blocks NF‐κB activation, while MTX promotes extracellular adenosine accumulation to activate adenylyl cyclases and enhance intracellular cAMP production [54, 55]. Biologically, while pathways such as TNF, IL‐17, and MAPK represent downstream effector loops or upstream kinase conduits, NF‐κB serves as the master transcriptional engine governing inflammatory mediator expression, which is intrinsically counter‐regulated by cAMP signaling [56, 57, 58]. Consistently, gene ontology (GO) enrichment analysis showed that the regulated genes were involved in immune‐inflammatory processes, including leukocyte chemotaxis, neutrophil migration, cytokine receptor binding, chemokine activity, and receptor ligand activity (Figure 6e and Figure S26). These results indicate that MB@YW NPs reshape RA‐associated immune‐inflammatory transcriptional programs and point to the cAMP/NF‐κB axis as a potential key regulatory pathway underlying their anti‐inflammatory effects.

To further examine this potential cAMP/NF‐κB regulatory mechanism, we analyzed representative genes within these two interconnected pathways. Heatmap analysis of the NF‐κB signaling pathway showed that RA markedly increased the expression of multiple upstream receptors, adaptor molecules, and inflammatory mediators, including *Il1r1*, *Tlr4*, *Eda2r*, *Ripk1*, and *Tab3*, whereas MB@YW NPs treatment substantially attenuated their upregulation (Figure 6g). In contrast, genes associated with the cAMP signaling pathway, such as *Adcy3*, *Crebbp*, *Fos*, *Rac3*, and *Pik3r3*, were generally suppressed in untreated inflammatory RAW264.7 cells but were broadly restored after MB@YW NPs treatment (Figure 6h). This reciprocal transcriptional pattern, characterized by restoration of cAMP‐associated genes and concomitant suppression of NF‐κB‐related inflammatory genes, supports a mechanistic model in which MB@YW NPs alleviate RA inflammation by reactivating cAMP‐linked anti‐inflammatory transcriptional programs and attenuating NF‐κB‐centered pro‐inflammatory signaling.

## 3 Conclusion

Rheumatoid arthritis exemplifies a broader class of complex inflammatory diseases in which spatially confined pathology, dynamic biochemical signals, and heterogeneous therapeutic requirements collectively hinder precise intervention. Despite advances in responsive nanomedicine, achieving independent control over multiple therapeutic agents within a single platform, while simultaneously enabling real‐time feedback of their activation, remains a fundamental challenge.

Here, we address these challenges through the development of MB@YW NPs, a programmable triple‐locked nanotheranostic platform that seamlessly synchronizes spatial localization, chemically orthogonal multidrug liberation, and real‐time multichannel self‐reporting. Through coordinated pH‐, ROS/RNS‐, and light‐gated unlocking, the platform translates extracellular acidity, intracellular inflammatory cues, and external optical inputs into precisely regulated therapeutic outputs. Within this framework, HOCl selectively activates MB‐MTX to release MTX with concomitant NIR fluorescence turn‐on, whereas ONOO^−^ and subsequent light irradiation independently trigger the stepwise liberation of SSZ accompanied by blue fluorescence activation and quenching. This hierarchical design enables spatiotemporally resolved drug release together with real‐time visualization of each activation event.

As a consequence, MB@YW NPs effectively alleviate oxidative stress, restore bioenergetic balance, and remodel the inflammatory microenvironment. In collagen‐induced arthritis models, the platform markedly suppresses synovial inflammation, prevents progressive joint destruction, and outperforms corresponding monotherapies. Transcriptomic analyses further reveal that these therapeutic benefits are associated with coordinated modulation of the cAMP/NF‐κB signaling axis.

Looking toward future clinical translation, systematic evaluations of the long‐term biodistribution, degradation kinetics, and metabolic clearance pathways of these nanomaterials will be essential. Furthermore, exploring advanced light delivery modalities, such as optical fiber‐guided or arthroscopy‐assisted irradiation, will be critical to circumvent tissue penetration limitations and ensure effective photon delivery to deeper human joints. In summary, this work demonstrates that chemically orthogonal logic gating can fundamentally reshape how combination nanomedicines are designed and controlled, providing a conceptual framework for next‐generation precision nanomedicine capable of autonomous and feedback‐driven intervention in complex disease microenvironments.

## Author Contributions

**Jihong Liu**: conceptualization, methodology, investigation, validation, funding acquisition, Writing – original draft, Writing – review and editing. **Wendi Zhu**: writing – review and editing, conceptualization, methodology, formal analysis. **Shiyun Xu**: conceptualization, methodology, visualization, investigation, software. **Shichang Liu**: investigation. **Wenjun Tang**: formal analysis, project administration. **Xiaoyu Ma**: formal analysis, project administration. **Ting Yang**: visualization. **Haojian Yang**: visualization. **Hongtong Wang**: formal analysis. **Lu Cao**: data curation, resources. **Lijie Tan**: resources, data curation. **Qi Wang**: funding acquisition, supervision, writing – review and editing, writing – original draft.

## Funding

This work was supported by the National Key Research and Development Program of China (2024YFA1803501), NSFC (T2488302 and 22404056), Science and Technology Commission of Shanghai Municipality (24DX1400200).

## Conflicts of Interest

The authors declare no conflicts of interest.

## Supporting information




**Supporting File**: advs78405‐sup‐0001‐SuppMat.docx.

## Data Availability

The data that support the findings of this study are available from the corresponding author upon reasonable request.
