# Supplementary material for: Spatiotemporally Precise Rheumatoid Arthritis Therapy via a Triple‐Locked Cascaded Nanoplatform With Dual‐Channel Self‐Reporting
Source: Adv Sci (Weinh). 2026 Oct 22:e78405. Online ahead of print. doi: 10.1002/advs.78405 (PMC13691242; doi:10.1002/advs.78405)
Supplement: Supplementary file 1 [file ADVS-9999-e78405-s001.docx]

Supporting Information

Spatiotemporally Precise Rheumatoid Arthritis Therapy via a Triple-Locked Cascaded Nanoplatform with Dual-Channel Self-Reporting

*Jihong Liu,^+[a,b]^ Wendi Zhu,^+[c]^ Shiyun Xu,^[a]^ Shichang Liu,^[a]^ Wenjun Tang,^[a]^ Xiaoyu Ma,^[a]^ Ting Yang,^[a]^ Haojian Yang,^[a]^ Hongtong Wang,^[a]^ Lu Cao,^[d]^ Lijie Tan,^[c]^ and Qi Wang*^[a,b]^*

[a] Key Laboratory for Advanced Materials and Joint International Research Laboratory of Precision Chemistry and Molecular Engineering, Shanghai Key Laboratory of Functional Materials Chemistry, Feringa Nobel Prize Scientist Joint Research Center, Institute of Fine Chemicals, Frontiers Science Center for Materiobiology and Dynamic Chemistry, School of Chemistry and Molecular Engineering, East China University of Science and Technology, Shanghai 200237, China

[b] Center of Photosensitive Chemicals Engineering, East China University of Science and Technology, Shanghai 200237, China

[c] Department of Thoracic Surgery, Zhongshan Hospital, Fudan University, Shanghai 200032, China

[d] Department of Orthopedic Surgery, Zhongshan Hospital, Fudan University, Shanghai 200032, China

E-mail: wangqi@ecust.edu.cn

**Table of Contents**

[**Experimental Procedures**	4](#_Toc241416058)

[**Results and Discussion**	13](#_Toc241416059)

[**Synthesis Route**	13](#_Toc241416060)

[**Scheme S1.**	13](#_Toc241416061)

[**Figure S1.**	20](#_Toc241416062)

[**Figure S2.**	21](#_Toc241416063)

[**Figure S4.**	23](#_Toc241416064)

[**Figure S5.**	24](#_Toc241416065)

[**Figure S6.**	25](#_Toc241416066)

[**Figure S7.**	26](#_Toc241416067)

[**Figure S8.**	27](#_Toc241416068)

[**Figure S9.**	28](#_Toc241416069)

[**Figure S12.**	31](#_Toc241416070)

[**Figure S13.**	32](#_Toc241416071)

[**Figure S14.**	33](#_Toc241416072)

[**Figure S15.**	34](#_Toc241416073)

[**Figure S16.**	35](#_Toc241416074)

[**Figure S17.**	36](#_Toc241416075)

[**Figure S18.**	37](#_Toc241416076)

[**Figure S19.**	38](#_Toc241416077)

[**Figure S20.**	39](#_Toc241416078)

[**Figure S21.**	40](#_Toc241416079)

[**Figure S22.**	41](#_Toc241416080)

[**Figure S23.**	42](#_Toc241416081)

[**Figure S24.**	43](#_Toc241416082)

[**Figure S25.**	44](#_Toc241416083)

[**Figure S26.**	45](#_Toc241416084)

[**Figure S27.**	46](#_Toc241416085)

[**Figure S28.**	47](#_Toc241416086)

[**Figure S29.**	48](#_Toc241416087)

[**Figure S30.**	49](#_Toc241416088)

[**Figure S31.**	50](#_Toc241416089)

[**Figure S32.**	51](#_Toc241416090)

[**Figure S33.**	52](#_Toc241416091)

[**Figure S34.**	53](#_Toc241416092)

[**Figure S35.**	54](#_Toc241416093)

[**Figure S36.**	55](#_Toc241416094)

[**Figure S37.**	56](#_Toc241416095)

[**Figure S38.**	57](#_Toc241416096)

[**Figure S39.**	58](#_Toc241416097)

# **Experimental Procedures**

**Materials and Instruments**

Unless special stated, all solvents and chemicals were purchased from commercial suppliers in analytical grade and used without further purification. ^1^H NMR spectra were obtained at 400 MHz using a Bruker Avance III spectrometer and ^13^C NMR spectra were recorded at 100 MHz. High resolution mass spectrometry data were obtained with a Q ExactiveTM UHMR spectrometer (Thermo Fisher Scientific). Absorption spectra were recorded on a UV-Visible spectrophotomer (UV-1900i, Shimadzu). Fluorescence spectra were obtained using fluorescence spectrophotometer (RF-6000, Shimadzu). Particle size was measured by dynamic light scattering (DLS) with an ALV light scattering apparatus. Zeta potential measurements were carried out on a Zetasizer Nano-ZSE instrument (Malvern, UK). pH assay was performed using a pH measuring instrument (SD50 HA kit, Mettler Toledo). High-performance liquid chromatography (HPLC) analysis was performed using a Shimadzu CBM-20A series system. Confocal imaging was performed on Leica SP8 high-resolution fluorescence microscope. Flow cytometry assay was performed on a flow cytometer (CytoFLEX LX, Beckman Coulter). *In vivo* fluorescence images were measured with a PE in vivo Professional Imaging System (IVIS Spectrum Imaging System). Transmission electron microscopy (TEM) images were captured on a JEOL-JEM-2100 transmission electron microscope. Gel permeation chromatography (GPC) analysis was performed on a Shimadzu CBM-20A series system. Micro-computed tomography (Micro-CT) imaging was carried out on a Bruker Skyscan 1276 system.

**Fabrication of the MB@YW NPs Theranostic Nanoplatform**

The theranostic nanoplatform (designated as MB@YW NPs), comprising the pH-responsive polymer PEG-PND co-encapsulating the prodrugs MB-MTX and YW-SSZ, was fabricated via a customized oil-in-water (O/W) emulsion solvent evaporation technique. Briefly, MB-MTX (0.2 mg) and YW-SSZ (0.2 mg) were co-dissolved in 200 μL of dichloromethane (DCM). This organic phase was subsequently injected rapidly into 1.8 mL of an aqueous solution containing PEG-PND (10 mg). The mixture was then emulsified using an ultrasonic cell disruptor at 20% amplitude for 5 min in an ice-water bath to yield a homogeneous O/W emulsion. Finally, DCM was removed by rotary evaporation under reduced pressure, yielding a uniform aqueous dispersion of MB@YW NPs. The concentration of MB@YW NPs was expressed as the equivalent concentration of MB-MTX, and the final MB-MTX prodrug concentration in the dispersion was 100 μg/mL.

**Hydrodynamic Size and Zeta Potential Analysis**

To evaluate the pH-responsive behavior, a 100 μL aliquot of the pristine MB@YW NPs dispersion was introduced into 1400 μL of buffer solutions at pH 5.0 and pH 7.4, respectively. After a 30 min incubation to ensure complete equilibration, the hydrodynamic diameters and Zeta potentials of the nanoparticles were systematically measured using a Malvern Zetasizer Nano ZS90 dynamic light scattering (DLS) instrument.

**Morphological Characterization**

The morphology of the nanoparticles was characterized by transmission electron microscopy (TEM). Briefly, 10–15 μL of the nanoparticle suspension was drop-cast onto a copper grid. After a 30 min settling period, the excess liquid was carefully wicked away using filter paper. The samples were allowed to dry naturally at room temperature prior to TEM observation. To visually corroborate the pH-triggered matrix dissociation, MB@YW NPs were pre-incubated in buffer solutions (pH 5.0 and 7.4) for 30 min to ensure complete acid-triggered degradation before subsequent TEM imaging and DLS analysis.

**Colloidal Stability Evaluation**

To assess the colloidal stability under simulated physiological conditions, MB@YW NPs were dispersed in a buffer solution at pH 7.4. The hydrodynamic size and polydispersity index (PDI) were continuously monitored using the Malvern Zetasizer Nano ZS90 instrument at 6 h intervals over a prolonged period of 66 h.

**Spectroscopic Evaluations and Stimuli-Responsiveness**

Following a 30 min pre-incubation of MB@YW NPs under acidic conditions (to trigger the “first-lock”), the fluorescence emission spectra were recorded upon the addition of HOCl and ONOO⁻ (the “second-lock”), respectively. Furthermore, the selectivity profiles, photostability, and linear correlation coefficients were systematically evaluated. To assess the light-gated activation (the “third-lock”), the sample which have undergone complete acid pre-treatment and ONOO⁻-mediated cleavage, was subjected to continuous irradiation using a 125 W medium-pressure xenon lamp equipped with a 400-650 nm bandpass filter. The fluorescence spectra were subsequently recorded at 2 min intervals over a total irradiation period of 30 min for MB@YW NPs.

**Cell Lines**

The RAW264.7 macrophages was purchased from the Institute of Cell Biology (Shanghai, China). RAW264.7 macrophages cells were cultured in DMEM (GIBCO/Invitrogen, Camarillo, CA, USA) supplemented with 10% fetal bovine serum (FBS, Biological Industry, Kibbutz Beit Haemek, Israel) and 1% penicillin-streptomycin (10,000 U/mL penicillin and 10 mg/mL streptomycin, Solarbio Life Sciences, Beijing, China). The cells were incubated at 37 °C in a humidified atmosphere containing 5% CO_2_.

**Intracellular Uptake of MB@YW NPs**

RAW264.7 macrophages were seeded in glass-bottom confocal dishes at a density of 1 × 10^5^ cells/mL and allowed to adhere overnight. To establish an *in vitro* inflammatory microenvironment, the cells were stimulated with lipopolysaccharide (LPS, 100 ng/mL) for 4 h. The LPS-containing medium was then discarded, and the cells were washed thrice with PBS.

Subsequently, the cells were incubated with MB@YW NPs (40 μM, based on MB-MTX equivalent) dispersed in mildly acidic culture medium (pH 6.5, tailored to mimic the synovial acidity of inflamed joints) for 2, 4, and 6 h, respectively. Unstimulated RAW264.7 cells treated with MB@YW NPs under identical conditions served as the control. Post-incubation, the nanoparticle solution was removed, and the cells were washed thrice with PBS prior to imaging via a confocal laser scanning microscope (CLSM, Leica TCS SP8). The imaging parameters were set as follows: Blue channel: λ_ex_ = 405 nm, λ_em_ = 420-550 nm; Red channel: λ_ex_ = 638 nm, λ_em_ = 640-800 nm. For quantitative analysis, RAW264.7 cells were seeded in 6-well plates (1 × 10^5^ cells/mL), cultured for 6 h to adhere, and subjected to the same treatments. After washing thrice with PBS, the cells were harvested via trypsinization, centrifuged (3000 rpm for 5 min, repeated thrice for washing), resuspended, and filtered through a 40 μm cell strainer. The intracellular fluorescence intensity was finally quantified using a flow cytometer (Beckman Coulter).

**Cellular Internalization of Different Nanoparticle Formulations**

Following the aforementioned seeding and LPS-stimulation (100 ng/mL, 4 h) protocols, RAW264.7 cells were incubated with various nanoformulations, including YW NPs, MB NPs, and MB@YW NPs, all dispersed in the mildly acidic medium. Specifically, YW NPs were administered at a concentration equivalent to 40 μM YW-SSZ, whereas MB NPs and MB@YW NPs were administered at concentrations equivalent to 40 μM MB-MTX for 6 h. Unstimulated cells incubated with MB@YW NPs were utilized as the control. After washing thrice with PBS, the cells were imaged using CLSM with the parameters described above. For quantitative evaluation, cells cultured in 6-well plates were treated identically, harvested, and analyzed via flow cytometry following the standardized procedures detailed in the previous section.

**Dynamic Monitoring of HOCl and ONOO⁻ Fluctuations**

To dynamically monitor HOCl and ONOO⁻ fluctuations, adherent RAW264.7 cells cultured in glass-bottom dishes were pre-treated with distinct exogenous ROS and RNS donors or inflammatory stimuli: HOCl (10 μM, 15 min), SIN-1 (an ONOO⁻ donor, 100 μM, 30 min), and LPS (100 ng/mL, 4 h), respectively. Untreated RAW264.7 cells served as the baseline control. Following thorough washing with PBS (thrice) to remove residual stimuli, the stimulated cells were incubated with MB@YW NPs (40 μM MB-MTX equivalent) in the mildly acidic medium for 6 h. The intracellular fluorescence signals were then mapped using CLSM under the previously established imaging parameters.

***In Vitro* Light-Triggered Cleavage Evaluation**

RAW264.7 cells were seeded and stimulated with LPS (100 ng/mL, 4 h) in glass-bottom dishes. After washing thrice with PBS, the cells were incubated with MB@YW NPs (40 μM MB-MTX equivalent) for 6 h, with unstimulated cells acting as the control. Post-incubation, the cells were washed thrice with PBS, and the medium was replaced with fresh DMEM. To evaluate the light-gated activation (the “third-lock”), the cells were subjected to external light irradiation (400-650 nm) for pre-determined durations (0, 5, and 10 min). The real-time fluorescence variations were immediately recorded via CLSM.

**Assessment of Intracellular ROS Scavenging Capacity**

RAW264.7 cells were cultured in glass-bottom dishes and stimulated with LPS (100 ng/mL) for a prolonged period of 12 h to induce robust intracellular ROS accumulation. Following PBS washing, the cells were separately incubated with YW NPs, MB NPs, and MB@YW NPs (40 μM equivalent) for a total of 24 h, during which light irradiation was applied. Unstimulated cells treated with MB@YW NPs served as the control. Subsequently, the cells were washed thrice with PBS and stained with the ROS-sensitive fluorescent probe DCFH-DA (5 μM) for 30 min. After a final triple wash with PBS, the intracellular ROS scavenging efficacy was visualized via CLSM. Green channel (DCFH-DA): λ_ex_ = 488 nm, λ_em_ = 490-550 nm.

**Evaluation of Intracellular ATP Restoration**

Following the identical 12 h LPS stimulation and washing protocol, RAW264.7 cells were incubated with YW NPs, MB NPs, and MB@YW NPs (40 μM equivalent) for 24 h, during which light irradiation was applied. Unstimulated cells incubated with MB@YW NPs were used as the control. To evaluate mitochondrial ATP homeostasis, the cells were washed thrice with PBS and subsequently incubated with a reported ATP-specific fluorescent probe, UDP (25 μM), for 30 min. Post-staining, the cells were washed thrice with PBS and imaged using CLSM. Purple channel (UDP): λ_ex_ = 514 nm, λ_em_ = 575-650 nm.

**Quantification of Pro-inflammatory Cytokines**

RAW264.7 cells were seeded and stimulated with LPS (100 ng/mL) for 12 h. After discarding the medium and washing thrice with PBS, the cells were incubated with MB@YW NPs (40 μM MB-MTX equivalent) for 24 h, during which light irradiation was applied. Unstimulated cells treated with MB@YW NPs were utilized as the baseline control. Upon completion of the incubation, the cell culture supernatants were carefully collected and centrifuged at 3000 rpm for 20 min to remove cellular debris. The concentrations of secreted pro-inflammatory cytokines in the supernatants were systematically quantified using commercial Enzyme-Linked Immunosorbent Assay (ELISA) kits. The optical density (OD) of each well was measured at 450 nm using a microplate reader to calculate the final cytokine concentrations.

***In Vitro* Cytotoxicity Assay**

The standard Cell Counting Kit-8 (CCK-8) assay was employed to evaluate the *in vitro* cytotoxicity of MB@YW NPs. RAW264.7 cells were seeded in 96-well plates at a density of 5 × 10^4^ cells/well (100 μL/well) and cultured for 6 h to allow attachment. Subsequently, the cells were treated with varying concentrations of MB@YW NPs (0, 6, 12, 18, 24, 30, 36 and 42 μg/mL) and incubated in the dark for 24 h. Six replicate wells were set for each group. Following incubation, 100 μL of fresh DMEM containing 10% CCK-8 reagent was added to each well, and the plates were further incubated for 2 h. The optical density (OD) at 450 nm was recorded using a microplate reader. Cell viability was calculated using the following equation:

Cell viability (%) = [(OD_sample_ – OD_blank_) / (OD_control_ – OD_blank_)] × 100%

**Hemocompatibility Evaluation**

The hemocompatibility of the nanoplatform was assessed via a standard hemolysis assay. Briefly, 250 μL of a 4% fresh rabbit red blood cell (RBC) suspension was mixed with 250 μL of MB@YW NPs at various concentrations (30, 60, 90, 120, 150, and 240 μg/mL). The mixtures were incubated at room temperature for 2 h and 4 h, respectively. Ultrapure water and 0.9% NaCl solution were used to replace the nanoparticle suspension as the positive and negative controls, respectively. Post-incubation, the samples were centrifuged at 10,000 rpm for 10 min at 4 °C. The supernatants were photographed, and their absorbance at 570 nm was measured using a microplate reader. The hemolysis ratio was calculated as follows:

Hemolysis (%) = [(OD_sample_ − OD_negative_) / (OD_positive_ − OD_negative_)] × 100%

**Animals**

Female Sprague-Dawley (SD) rats (10 weeks old) were purchased from SPF Biotechnology Co., Ltd. (Shanghai, China). Female DBA/1 mice (6 weeks old) were obtained from GemPharmatech Co., Ltd. (Jiangsu, China). Ethical approval for the animal experiments was obtained prior to the research from East China University of Science and Technology Animal Studies Committee, and the assigned approval number was ECUST-2024-072.

**Establishment of the SD Rat Arthritis Model and *In Vivo* Imaging**

The SD rats were randomly assigned to three groups (*n* = 3): healthy, unilateral arthritis, and bilateral arthritis. To induce arthritis, rats in the bilateral group received intra-articular injections of 300 μL of carrageenan solution (5 mg/mL) into both hind limbs for 2 consecutive days. The unilateral group received the carrageenan injection only in the right hind limb, with an equal volume of PBS injected into the left hind limb. The healthy group received PBS injections in both hind limbs. Prior to administration, MB@YW NPs were diluted in sterile PBS and subjected to brief vortexing to ensure a uniform and stable suspension. One day post-modeling, MB@YW NPs (5 mg/kg, based on MB-MTX equivalent) were administered intravenously via the tail vein. *In vivo* fluorescence imaging (λ_ex_ = 620 nm, λ_em_ = 710 nm) was continuously performed at 0.5, 1, 2, 3, 4, and 6 h post-injection using an IVIS Spectrum imaging system (PerkinElmer). At 6 h post-injection, the rats were euthanized, and major organs were excised for *ex vivo* imaging.

***Ex Vivo* Tissue Penetration Depth Study**

To assess the deep-tissue penetration of the nanoparticles, the ankle joints of both healthy and arthritic rats were excised at 6 h post-injection. The joint tissues were washed with ice-cold PBS, placed in glass-bottom dishes, and subjected to Z-stack scanning using a confocal laser scanning microscope (CLSM). The scanning interval was set at 10 μm. The imaging parameters for the blue (YW-SSZ) and red (MB-MTX) channels were consistent with the *in vitro* settings. 3D reconstructive data analyses were performed using LAS X software.

**Establishment of the Collagen-Induced Arthritis (CIA) Mouse Model**

Female DBA/1 mice (6 weeks old) were obtained from GemPharmatech Co., Ltd. (Jiangsu, China) and acclimatized for one week. The highly aggressive CIA model was established following standard protocols. Briefly, an immunization emulsion was prepared by mixing immunization-grade bovine type II collagen (CII, 4 mg/mL dissolved in 0.05 N acetic acid) with Complete Freund’s Adjuvant (CFA, 2 mg/mL) at a 1:1 volume ratio, followed by rigorous emulsification in an ice bath. On Day 0, the 7-week-old mice were anesthetized and subcutaneously injected with 100 μL of the CII-CFA emulsion at the base of the tail. On Day 20, a booster immunization was administered using an emulsion of CII and Incomplete Freund’s Adjuvant (IFA). Erythema and swelling of the hind paws typically emerged by Day 21, and the CIA model was fully established and confirmed by Day 28.

***In Vivo* Therapeutic Regimen and Spatiotemporal Imaging**

Beginning on Day 30 post-primary immunization, the CIA mice were randomly divided into treatment groups and intravenously injected with PBS (100 μL), YW NPs, MB NPs, or MB@YW NPs (5 mg/kg MB-MTX/YW-SSZ equivalent). The treatments were administered every 3 days for a total of 5 doses, accompanied by 30 min of targeted localized light irradiation (400-650 nm, 80 mW cm^-2^) after each injection. Healthy DBA/1 mice receiving equal volumes of PBS served as the absolute control. Throughout the therapeutic window, body weights, right hind paw thicknesses, and clinical arthritis scores were meticulously recorded every 2 days. Photographic documentation was performed post-treatment.

To validate the *in vivo* tracking capability, on Day 48, mice from all groups were intravenously injected with MB@YW NPs (100 μL) and imaged 6 h post-injection using the IVIS Spectrum system. Furthermore, to monitor the light-gated “On-to-Off” decoding, the PBS-treated RA group was subjected to external light irradiation (400-650 nm, 80 mW cm^-2^), with fluorescence signals recorded at 10 min intervals over a 30 min period (Red channel: λ_ex_ = 620 nm, λ_em_ = 710 nm; Blue channel: λ_ex_ = 420 nm, λ_em_ = 520 nm).

**Clinical Evaluation of Arthritis Severity**

To evaluate the therapeutic efficacy of MB@YW NPs against arthritis, the clinical severity of arthritis in all four paws of each mouse was monitored and scored in a blinded manner according to standard macroscopic scoring criteria. Score 0: No evidence of erythema and swelling occurred; Score 1: Erythema and mild swelling appeared; Score 2: Erythema and mild swelling extended from the ankle to the tarsals; Score 3: Erythema and moderate swelling extended from the ankle to metatarsal joints; Score 4: Erythema and severe swelling encompassed the ankle, foot, and digits, or ankylosis of the limb. The final clinical arthritis score of each mouse was calculated as the cumulative sum of all four individual limb scores (yielding a maximal possible score of 16 per mouse).

**Micro-CT Imaging for Bone Morphological Assessment**

Upon completion of the therapeutic regimen (Day 48), the mice were euthanized. The hind paws were harvested, fixed in 4% paraformaldehyde (PFA) overnight, and stored in PBS. The preservation of osteochondral structural integrity and bone microarchitecture was systematically evaluated using a high-resolution Micro-CT scanner (Bruker Skyscan 1276).

***Ex Vivo* Evaluation of ROS Scavenging and Cytokine Neutralization**

Post-treatment, the hind paws were harvested, and the soft tissues were carefully dissected. The joint tissues were homogenized in ice-cold PBS to extract the synovial fluid. After centrifugation (3000 rpm, 15 min), the supernatant was collected. To quantify ROS scavenging, the supernatant was incubated with DCFH-DA for 15 min, and the fluorescence intensity was measured using a fluorescence spectrometer. Concurrently, the concentrations of pro-inflammatory cytokines in the synovial fluid were quantified using commercial ELISA kits.

**Histological, Immunohistochemical, and Immunofluorescence Analyses**

For histological evaluations, the excised hind paws were fixed in 4% PFA overnight and subsequently decalcified in a 10% EDTA solution for 14 days. The decalcified tissues were paraffin-embedded and sectioned. The sections were subjected to Hematoxylin and Eosin (H&E) and Safranin O-Fast Green (SO-FG) staining to assess synovial hyperplasia and cartilage erosion. Furthermore, immunohistochemistry (IHC) for TNF-α and IL-6, as well as immunofluorescence (IF) staining for TGF-β and IL-10, were performed. The stained sections were captured and analyzed using bright-field microscopy and CLSM.

**Transcriptomic (RNA-seq) Analysis**

RAW264.7 cells (1 × 10^5^ cells/mL) were cultured in glass-bottom dishes and stimulated with LPS (100 ng/mL) for 24 h. Subsequently, the cells were treated with MB@YW NPs (40 μM MB-MTX equivalent) for another 24 h. Unstimulated and untreated cells served as controls. Post-treatment, the cells were washed thrice with PBS and immediately lysed using Trizol reagent in an ice bath. The lysates were snap-frozen in liquid nitrogen and stored at -80 °C. Total RNA extraction, library construction, and deep RNA sequencing (RNA-seq) were performed by Novogene Co., Ltd. (Beijing, China).

***In Vivo* Systemic Biosafety Evaluation**

Following the therapeutic regimen, major organs (heart, liver, spleen, lung, and kidneys) were harvested, fixed in 4% PFA, paraffin-embedded, and stained with H&E to evaluate potential systemic toxicity and off-target apoptosis. Additionally, whole blood was collected in anticoagulant tubes and stored at 4 °C overnight for standard hematological analysis. For serum biochemical analysis (hepatic and renal function panels), the blood was allowed to clot at room temperature for 4 h, followed by centrifugation at 12,000 rpm for 20 min to obtain the serum, which was immediately stored at -80 °C until analysis (*n* = 3).

**Statistical Analysis**

All quantitative data are presented as the mean ± standard deviation (SD). Unless otherwise specified, *in vitro* experiments were performed in independent triplicates (*n* = 3), and *in vivo* experiments utilized 3 mice per group (*n* = 3). Statistical significance for two-group comparisons was evaluated using the two-tailed Student’s t-test. For multiple-group comparisons involving a single independent factor, one-way analysis of variance (ANOVA) followed by Tukey’s *post-hoc* test was applied. For longitudinal *in vivo* fluorescence tracking across multiple time points, repeated-measures (RM) two-way ANOVA was conducted, followed by Sidak’s or Tukey’s post-hoc multiple-comparison tests as appropriate. Statistical significance is denoted in the figures and figure legends as **P* < 0.05, ***P* < 0.01, ****P* < 0.001, and *****P* < 0.0001.

# **Results and Discussion**

## **Synthesis Route**

**Scheme S1. The synthesis route of MB-MTX, YW-TDP, and PEG-PND.**

**Synthesis of MB-Cl**

To a 250 mL two-neck round-bottom flask, methylene blue (1.60 g, 5.0 mmol) and sodium carbonate (1.06 g, 10.0 mmol) were dissolved in ultrapure water, followed by the addition of dichloromethane (DCM, 15 mL). The biphasic mixture was stirred under a nitrogen atmosphere for 5 min until the solution became clear. Subsequently, an aqueous solution of sodium dithionite (1.74 g, 10.0 mmol) was slowly injected into the mixture. The reaction was allowed to proceed at 40 °C for 30 min. Upon observing the solution turn pale yellow, the heating was discontinued. After cooling in a water bath for 10 min, a solution of bis(trichloromethyl) carbonate (triphosgene, 890.24 mg, 3.0 mmol) in DCM was added dropwise into the mixture under an ice-water bath. Following complete addition, the ice-water bath was removed, and the reaction mixture was stirred at room temperature for 1 h. The resulting mixture was then extracted with DCM (3 × 50 mL). The combined organic layers were dried over anhydrous Na₂SO₄, filtered, and concentrated under reduced pressure. The crude residue was purified by silica gel column chromatography (petroleum ether/ethyl acetate = 4:1, v/v) to afford the target compound as a white solid (333.9 mg, yield: 31.9%). ^1^H NMR (400 MHz, DMSO-*d*_6_, ppm): *δ* 7.43 (d, *J* = 8.9 Hz, 2H, Ar-H), 6.79 (d, *J* = 2.8 Hz, 2H, Ar-H), 6.71 (dd, *J_1_* = 2.9 Hz, *J_2_* = 6.0 Hz, 2H, Ar-H), 2.92 (s, 12H, -C***H***_3_).

**Synthesis of MB-BTD**

To a 100 mL two-neck round-bottom flask, 1,4-butanediamine (132.2 mg, 1.5 mmol) and dichloromethane (DCM, 10 mL) were added sequentially under a nitrogen atmosphere. After stirring until complete dissolution, solid sodium carbonate (158.9 mg, 1.5 mmol) was introduced to the solution, and the resulting suspension was stirred for 5 min. Subsequently, a solution of MB-Cl (174.5 mg, 0.5 mmol) dissolved in DCM was slowly injected into the flask. The reaction mixture was then allowed to stir at room temperature for 2 h. Upon completion of the reaction, the insoluble sodium carbonate was removed by filtration. The filtrate was concentrated under reduced pressure to remove the solvent. The obtained crude residue was purified by silica gel column chromatography (DCM/methanol = 20:3, v/v) to afford the desired product as a pale blue solid (102.7 mg, yield: 50.7%). ^1^H NMR (400 MHz, DMSO-*d*_6_, ppm): *δ* 7.26 (d, *J* = 8.8 Hz, 2H, Ar-H), 6.70 (d, *J* = 2.9 Hz, 2H, Ar-H), 6.65 (dd, *J_1_* = 2.9 Hz, *J_2_* = 5.9 Hz, 2H, Ar-H), 6.07 (s, 1H, -N***H***CH_2_-), 3.00 (t, *J* = 5.8 Hz, 2H, -C***H***_2_NH_2_-), 2.89 (m, 14H, 2H for -NHC***H***_2_-, 12H for -C***H***_3_), 1.38 (m, 6H, 2H for -CH_2_N***H***_2_-, 2H for -C***H***_2_CH_2_-, 2H for -CH_2_C***H***_2_-).

**Synthesis of MB-MTX**

To a 100 mL two-neck round-bottom flask under a nitrogen atmosphere, methotrexate (MTX, 170.42 mg, 0.375 mmol) and triethylamine (TEA, 50.6 mg, 0.5 mmol) were added sequentially and dissolved in anhydrous *N,N*-dimethylformamide (DMF). After stirring until complete dissolution, 4-dimethylaminopyridine (DMAP, 36.7 mg, 0.3 mmol) and *N,N’*-dicyclohexylcarbodiimide (DCC, 61.9 mg, 0.3 mmol) were successively introduced into the flask under an ice-water bath. The mixture was stirred for 5 min until a clear solution was obtained. Subsequently, a solution of MB-BTD (100 mg, 0.25 mmol) in anhydrous DMF was slowly added to the reaction mixture. Following complete addition, the ice-water bath was removed, and the reaction was allowed to proceed at 35 °C for 24 h. Upon completion of the reaction, the solvent (DMF) was partially removed under reduced pressure. The concentrated residue was then purified by preparative thin-layer chromatography (prep-TLC) using a solvent mixture of dichloromethane and methanol (DCM/MeOH = 3:1, v/v) as the eluent. The resulting residue was dried in vacuo to afford the desired product as a green solid (100.3 mg, yield: 48.0%). ^1^H NMR (400 MHz, DMSO-*d*_6_, ppm): *δ* 8.53 (s, 1H, -CHN***H***C-), 8.11 (d, *J* = 8.0 Hz, 1H, Ar-H), 7.96 (d, *J* = 8.1 Hz, 1H, Ar-H), 7.85 (s, 1H, -CH_2_N***H***-), 7.70 (d, *J* = 8.8 Hz, 3H, Ar-H), 7.24 (d, *J* = 8.8 Hz, 2H, Ar-H), 6.78 (d, *J* = 8.8 Hz, 2H, Ar-H), 6.67 (d, *J* = 3.3 Hz, 2H, Ar-H), 6.64 (d, *J* = 8.8 Hz, 4H, Ar-N***H***_2_), 5.98 (s, 1H, -N***H***CH_2_-), 4.76 (s, 2H, -NC***H***_2_-), 4.33 (t, *J* = 9.2 Hz, 1H, -CH_2_C***H***NH-), 3.19 (s, 3H, -NC***H***_3_), 2.99 (m, 4H, -C***H***_2_C***H***_2_-), 2.85 (s, 12H, -NC***H***_3_-), 1.62 (m, 4H, -C***H***_2_C***H***_2_-), 1.34 (m, 4H, -C***H***_2_C***H***_2_-). ^13^C NMR (100 MHz, DMSO-*d*_6_, ppm): *δ* 171.91, 169.65, 166.40, 163.17, 155.64, 153.78, 151.24, 149.54, 148.91, 146.56, 133.58, 129.39, 128.98, 127.82, 121.91, 121.65, 111.64, 111.38, 110.77, 53.36, 52.69, 49.96, 40.71, 38.89, 32.00, 27.54, 26.92, 25.87, 24.75. High-resolution mass spectrometry (ESI positive ion mode for [M - H]^-^): Calcd. for C_41_H_49_O_5_N_13_S: 834.3627; Found: 834.3613.

**Synthesis of YW-Br**

To a 250 mL two-neck round-bottom flask, 5-bromoacetyl-2-hydroxybenzaldehyde (243.05 mg, 1.0 mmol) was added and dissolved in dichloromethane (DCM). 2-aminobenzothiol (125.19 mg, 1.0 mmol) and the catalyst, 3,6-di(pyridin-2-yl)-1,2,4,5-tetrazine (1 mg, 0.004 mmol), were successively introduced. The resulting mixture was stirred and irradiated with a white light lamp under an open-air atmosphere at room temperature for 2 h. Upon completion of the reaction, the mixture was filtered to remove insoluble by-products. The filtrate was subsequently concentrated under reduced pressure. The crude residue was purified by silica gel column chromatography, eluting with a mixture of petroleum ether and ethyl acetate (6:1, v/v), to afford the target compound as a white solid (106.9 mg, yield: 30.7%). ^1^H NMR (400 MHz, CDCl_3_, ppm): *δ* 8.43 (s, 1H, Ar-H), 8.02 (t, *J* = 7.5 Hz, 2H, Ar-H), 7.97 (d, *J* = 8.0 Hz, 1H, Ar-H), 7.56 (t, *J* = 8.3 Hz, 1H, Ar-H), 7.47 (t, *J* = 8.2 Hz, 1H, Ar-H), 7.19 (d, *J* = 6.4 Hz, 1H, Ar-H), 4.44 (s, 2H, -C***H***_2_-).

**Synthesis of YW-TDP**

To a dry 100 mL two-neck round-bottom flask, YW-Br (193.8 mg, 0.56 mmol) and 4-(hydroxymethyl)phenylboronic acid pinacol ester (110.0 mg, 0.47 mmol) were added and dissolved in anhydrous tetrahydrofuran (THF). Subsequently, a solution of triphenylphosphine (PPh₃, 183.6 mg, 0.7 mmol) in anhydrous THF was slowly added dropwise to the mixture under an ice-water bath. After stirring for 15 min, diisopropyl azodicarboxylate (DIAD, 141.6 mg, 0.7 mmol), dissolved in anhydrous THF, was added. The ice-water bath was then removed, and the reaction was allowed to proceed at room temperature for 24 h. Upon completion of the reaction, the solvent was removed under reduced pressure. The crude residue was purified by silica gel column chromatography, eluting with a mixture of petroleum ether and ethyl acetate (5:1, v/v), to afford the target compound as a white solid (85 mg, yield: 27%). ^1^H NMR (400 MHz, CDCl_3_, ppm): *δ* 9.17 (s, 1H, Ar-H), 8.11 (d, *J* = 7.9 Hz, 1H, Ar-H), 8.05 (d, *J* = 8.6 Hz, 1H, Ar-H), 7.82 (m, 3H, Ar-H), 7.48 (m, 3H, Ar-H), 7.34 (t, *J* = 77.9 Hz, 1H, Ar-H), 7.13 (d, *J* = 9.0 Hz, 1H, Ar-H), 5.38 (s, 2H, -C***H***_2_O-), 4.55 (s, 2H, -C***H***_2_Br), 1.29 (s, 12H, -C***H***_3_).

**Synthesis of YW-SSZ**

To a dry 100 mL two-neck round-bottom flask, sulfasalazine (127.5 mg, 0.32 mmol) dissolved in anhydrous *N,N*-dimethylformamide (DMF) was added, followed by the addition of potassium carbonate (K₂CO₃, 88.5 mg, 0.64 mmol). The mixture was stirred for 5 min. Subsequently, YW-TDP (120.5 mg, 0.21 mmol) was introduced into the flask. The reaction mixture was stirred at room temperature for 6 h while being protected from light. The mixture was first diluted with ethyl acetate (EtOAc), and then washed with brine (3 × 200 mL) to thoroughly remove the DMF. The organic phase was collected and dried over anhydrous sodium sulfate (Na₂SO₄). After filtration, the solvent was removed under reduced pressure. The crude residue was purified by silica gel column chromatography (DCM/MeOH = 24:1, v/v) to afford the desired product as an orange solid (47.3 mg, yield: 25.2%). ^1^H NMR (400 MHz, CDCl_3_, ppm): *δ* 9.16 (s, 1H, -N***H***-), 8.13 (d, *J* = 8.3 Hz, 1H, Ar-H), 8.08 (d, *J* = 9.6 Hz, 1H, Ar-H), 7.92-7.86 (m, 4H, Ar-H), 7.82-7.77 (m, 5H, Ar-H), 7.56-7.49 (m, 4H, Ar-H), 7.41-7.33 (m, 6H, Ar-H), 7.17 (d, *J* = 9.2 Hz, 1H, Ar-H), 5.43 (s, 2H, -CC***H***_2_O-), 4.72 (s, 2H, -PhC***H***_2_-), 1.26 (s, 12H, -C***H***_3_-). ^13^C NMR (100 MHz, DMSO-*d*_6_, ppm): *δ* 197.96, 190.63, 171.69, 164.53, 161.68, 161.49, 159.83, 159.49, 153.87, 151.87, 145.70, 144.96, 135.92, 135.20, 134.75, 129.58, 128.38, 128.27, 127.95, 127.73, 126.83, 123.86, 123.19, 119.03, 114.40, 114.30, 84.22, 25.43, 25.15. High-resolution mass spectrometry (ESI positive ion mode for [M - H]^-^): Calcd. for C_46_H_39_O_9_N_5_BS_2_: 880.2288; Found: 880.2247.

**Synthesis of PEG-Br**

To a 100 mL single-neck flask, methoxypolyethylene glycol (Me-PEG-OH, Mw = 5000, 5.0 g, 1.0 mmol) and triethylamine (TEA, 0.16 g, 1.6 mmol) were added. Dry dichloromethane (DCM, 20 mL) was then introduced, and the mixture was stirred at room temperature for 10 min to ensure complete dissolution. Subsequently, 2-bromo-2-methylpropionyl bromide (1.5 g, 6.4 mmol), dissolved in dry DCM, was slowly added dropwise to the flask under an ice-water bath. After the addition was complete, the ice-water bath was removed, and the reaction mixture was stirred at room temperature for 5 h. Upon completion of the reaction, a portion of the solvent was removed under reduced pressure until the solution became a white viscous mass. The residue was then slowly added dropwise into 150 mL of cold diethyl ether (4 °C). After the precipitation of a large amount of white solid, the product was collected by suction filtration. The obtained white filter cake was dried under vacuum to yield 3.6 g of a pure white solid (yield: 71.2%). ^1^H NMR (400 MHz, CDCl_3_, ppm): *δ* 3.66 (m, 487H, -(OC***H***_2_C***H***_2_)_114_-), 3.39 (s, 3H, -OC***H***_3_), 1.94 (s, 6H, -C***H***_3_).

**Synthesis of PEG-PND**

The synthesis of the pH-activated polymer PEG-PND was meticulously conducted via Atom Transfer Radical Polymerization (ATRP), necessitating prior purification of key reagents. Initially, grayish-green copper(I) bromide (100.0 mg, 0.69 mmol) was placed in a 100 mL pear-shaped flask and sequentially washed with anhydrous ethanol, glacial acetic acid, ultrapure water, and anhydrous ethanol until the solid transformed to a pure white color. The purified CuBr was then collected by suction filtration, dried under vacuum, and stored under a nitrogen atmosphere. Concurrently, commercial 2-(diisopropylamino)ethyl methacrylate was purified by silica gel column chromatography using a 20:1 (v/v) mixture of petroleum ether and ethyl acetate.

For the polymerization, a dry 25 mL Schlenk tube was charged with PEG-Br (0.25 g, 0.05 mmol) and the purified 2-(diisopropylamino)ethyl methacrylate (DPAEMA, 0.85 g, 4.0 mmol). This mixture was then degassed by vacuum pumping for 2 h, followed by the addition of 4 mL of dry *N,N*-dimethylformamide (DMF), and sonication to ensure complete dissolution of both PEG-Br and DPAEMA. The Schlenk tube was subsequently placed in a Dewar flask, and the solution underwent one freeze-pump-thaw cycle (10 min) utilizing liquid nitrogen. Pentamethyldiethylenetriamine (PMDETA, 17.3 mg, 0.1 mmol) was then introduced, followed by two more freeze-pump-thaw cycles (2 × 10 min). Finally, the purified copper(I) bromide (CuBr, 14.4 mg, 0.1 mmol) was added, and the mixture subjected to three additional freeze-pump-thaw cycles (3 × 10 min). After the degassing cycles were complete, the liquid nitrogen was removed, and the reaction mixture was allowed to stir at 60 °C under a nitrogen atmosphere for 36 h. A noticeable color change from light blue to dark green, along with an increase in viscosity, indicated the progression of the reaction. Post-reaction, the crude mixture was purified by column chromatography using 100-mesh neutral aluminum oxide as the stationary phase and a 2:1 (v/v) mixture of tetrahydrofuran and *N,N*-dimethylformamide as the eluent to efficiently remove residual copper salts. The resulting pale yellow solution was concentrated by rotary evaporation to remove a portion of the solvent. The concentrated solution was then transferred to a 7000 Da regenerated cellulose (RC) dialysis bag and subjected to extensive dialysis, first against *N,N*-dimethylformamide (4 times) and then against ultrapure water (4 times). The purified solution was finally lyophilized to yield 146.3 mg of a pure white solid product. ^1^H NMR (400 MHz, CDCl_3_, ppm): *δ* 3.86 (s, 44H, -NC***H***_2_CH_2_O-), 3.65 (m, 521H, -(OC***H***_2_C***H***_2_)_114_-), 3.38 (s, 3H, -OC***H***_3_), 3.01 (s, 40H, -C***H***_2_C-), 2.63 (s, 40H, -NCH_2_C***H***_2_O-), 1.81 (s, 40H, -C***H***(CH_3_)_2_-), 1.25 (s, 6H, -C(C***H***_3_)_2_CO-), 1.02 (s, 240H, -CHC***H***_3_-), 0.90 (s, 60H, -CC***H***_3_).

**Figure S1. Morphological and physicochemical characterization of MB@YW NPs under varying pH conditions.** **(a)** Schematic illustration depicting the pH-responsive structural disassembly and cargo release of MB@YW NPs under acidic conditions (left), and representative TEM micrographs demonstrating the progressive morphological transition from intact spherical nanoparticles to disassembled aggregates upon incubation at pH 7.4, 6.5, and 5.0 (right; scale bars: 200, 100, and 50 nm). **(b)** Hydrodynamic size distributions of MB@YW NPs determined by DLS after incubation at pH 7.4, 6.5, and 5.0. **(c)** Zeta potentials of MB@YW NPs at pH 7.4, 6.5, and 5.0, showing a stepwise surface charge reversal under mildly acidic and acidic conditions. **(d)** Colloidal stability of MB@YW NPs monitored by hydrodynamic diameter over a 66 h period.

**Figure S2. Selectivity of MB@YW NPs toward HOCl under acidic conditions (pH 5.0).** Fluorescence response of MB@YW NPs upon treatment with HOCl (70 μM) compared to various biologically relevant competitive species: 1. Blank; 2. Na^+^ (10 mM); 3. K^+^ (10 mM); 4. Fe^3+^ (200 μM); 5. Mg^2+^ (200 μM); 6. Zn^2+^ (200 μM); 7. Ca^2+^ (200 μM); 8. ONOO^−^ (10 μM); 9. H_2_O_2_ (10 mM); 10. Vitamin C (200 μM); 11. GSH (200 μM); 12. O_2_^•−^ (100 μM); 13. Hcy (1 mM); 14. BSA (10 μg/mL); 15. CES2 (10 μg/mL); 16. HAase (10 μg/mL); 17. Pepsin (10 μg/mL); 18. HOCl (70 μM).

**Figure S3. Selectivity of MB@YW NPs toward HOCl under mildly acidic conditions (pH 6.5).** Fluorescence response of MB@YW NPs upon treatment with HOCl (70 μM) compared to various biologically relevant competitive species: 1. Blank; 2. Na^+^ (10 mM); 3. K^+^ (10 mM); 4. Fe^3+^ (200 μM); 5. Mg^2+^ (200 μM); 6. Zn^2+^ (200 μM); 7. Ca^2+^ (200 μM); 8. ONOO^−^ (10 μM); 9. H_2_O_2_ (10 mM); 10. Vitamin C (200 μM); 11. GSH (1 mM); 12. O_2_^•−^ (100 μM); 13. Hcy (1 mM); 14. BSA (10 μg/mL); 15. CES2 (10 μg/mL); 16. HAase (10 μg/mL); 17. Pepsin (10 μg/mL); 18. HOCl (70 μM).

**Figure S4. Spectroscopic characterization and responsiveness of the HOCl-activatable prodrug MB-MTX.** **(a)** Absorption spectra of MB-MTX upon treatment with HOCl (70 μM). **(b)** Fluorescence emission spectra showing the NIR signal turn-on of MB-MTX in response to HOCl (70 μM). **(c)** Linear correlation analysis between the fluorescence intensity at 699 nm and HOCl concentration (0-70 μM). **(d)** Specificity of MB-MTX toward HOCl over various biologically relevant species: 1. Blank; 2. Na^+^ (10 mM); 3. K^+^ (10 mM); 4. Fe^3+^ (200 μM); 5. Mg^2+^ (200 μM); 6. Zn^2+^ (200 μM); 7. Ca^2+^ (200 μM); 8. ONOO^−^ (10 μM); 9. H_2_O_2_ (10 mM); 10. Vitamin C (200 μM); 11. GSH (200 μM); 12. O_2_^•−^ (100 μM); 13. Hcy (1 mM); 14. BSA (10 μg/mL); 15. CES2 (10 μg/mL); 16. HAase (10 μg/mL); 17. Pepsin (10 μg/mL); 18. HOCl (70 μM). **(e)** pH-dependency of the fluorescence response of MB-MTX in the absence (gray) and presence (pink) of HOCl (70 μM). **(f)** Photostability of the HOCl-activated MB-MTX monitored at 699 nm under continuous irradiation. λ_ex_ = 625 nm, λ_em_ = 699 nm.

**Figure S5. Linear calibration curve of MB@YW NPs for ONOO⁻ sensing.** Plot of fluorescence intensity at 468 nm as a function of ONOO⁻ concentration.

**Figure S6. Schematic illustration of the chemically orthogonal activation pathways and structural transformations of the designed components.** The proposed reaction mechanisms detail: (1) the acid-responsive protonation of the PEG-PND polymeric carrier; (2) the selective oxidative cleavage of the specifically tailored urea linkage in the MB-MTX prodrug triggered by HOCl, leading to the release of MTX and the concurrent activation of the NIR fluorophore MB; and (3) Schematic illustration of the dual-input cascaded activation of the YW-SSZ prodrug. ONOO⁻-mediated oxidative deprotection of the pinacol boronate moiety first unmasks a blue-emitting phenolic species, which subsequently undergoes ESIPT-assisted deprotonation and photo-Favorskii rearrangement upon light irradiation, leading to SSZ liberation accompanied by blue fluorescence quenching.

**Figure S7. Spectroscopic characterization and responsiveness of the ONOO^−^-activatable prodrug YW-SSZ.** **(a)** Absorption spectra of YW-SSZ upon treatment with ONOO^−^ (5.25 μM). **(b)** Fluorescence emission spectra showing the blue signal turn-on of YW-SSZ in response to ONOO^−^ (5.25 μM). **(c)** Linear correlation analysis between the fluorescence intensity at 468 nm and ONOO^−^ concentration (0-5.25 μM). **(d)** Specificity of YW-SSZ toward ONOO^−^ over various biologically relevant species: 1. Blank; 2. Na^+^ (10 mM); 3. K^+^ (10 mM); 4. Fe^3+^ (200 μM); 5. Mg^2+^ (200 μM); 6. Zn^2+^ (200 μM); 7. Ca^2+^ (200 μM); 8. Vitamin C (200 μM); 9. GSH (200 μM); 10. HAase (10 μg/mL); 11. Pepsin (10 μg/mL); 12. BSA (10 μg/mL); 13. Pro (1 mM); 14. His (1 mM); 15. HOCl (70 μM); 16. O_2_^•−^ (100 μM); 17. H_2_O_2_ (10 mM); 18. ONOO^−^ (5.25 μM). **(e)** pH-dependency of the fluorescence response of YW-SSZ in the absence (gray) and presence (blue) of ONOO^−^ (5.25 μM), demonstrating stability across physiological pH ranges. **(f)** Photostability of the ONOO^−^-activated YW-SSZ monitored at 468 nm under continuous irradiation. λ_ex_ = 375 nm, λ_em_ = 468 nm.

**Figure S8. Light-triggered fluorescence response of activated prodrug YW-SSZ.** Time-dependent fluorescence decay of ONOO^−^-activated prodrug YW-SSZ under continuous light irradiation (80 mW cm^-2^, 400-650 nm).

**Figure S9. Comprehensive *in vitro* hemocompatibility and cytocompatibility assessment of MB@YW NPs.** **(a, b)** Visual observation of the hemolysis assay using 4% rabbit erythrocytes after 2 h and 4 h of incubation with MB@YW NPs at gradient concentrations (30–240 μg/mL). Deionized (DI) water and saline (0.9% NaCl) served as positive and negative controls, respectively. **(c, d)** Quantitative evaluation of the hemolysis percentages at (c) 2 h and (d) 4 h, demonstrating the excellent hemic safety of the nanoplatform. **(e)** Relative cell viability of RAW264.7 macrophages after 24 h of treatment with MB@YW NPs, as determined by the CCK-8 assay.

**Figure S10. Relative cell viability of RAW264.7 macrophages after various treatments, as determined by the CCK-8 assay.** Group 1: untreated control cells. Group 2: cells incubated with MB@YW NPs (42 μg/mL) without light irradiation. Group 3: cells incubated with MB@YW NPs (42 μg/mL) followed by 15 min light irradiation (400-650 nm, 80 mW cm^-2^). Group 4: cells incubated with MB@YW NPs (42 μg/mL) followed by 30 min light irradiation (400-650 nm, 80 mW cm^-2^). Group 5: cells exposed to irradiation (400-650 nm, 80 mW cm^-2^) for 15 min in the absence of MB@YW NPs. Group 6: cells exposed to irradiation (400-650 nm, 80 mW cm^-2^) for 30 min in the absence of MB@YW NPs.

**Figure S11. Real-time temperature monitoring of MB@YW NPs under continuous light irradiation.** **(a)** Representative infrared thermal images of pure acidic buffer (Ar1, left) and MB@YW NPs treated with ONOO⁻ in acidic buffer (Ar2, right) recorded at 1-min intervals during 0-29 min of continuous light irradiation (400-650 nm, 80 mW cm^-2^). **(b)** Temperature variation curves of the corresponding solutions during the 29-min irradiation process.

**Figure S12. Flow cytometric analysis of the cellular internalization of MB@YW NPs. (a)** Time-dependent internalization of MB@YW NPs in quiescent and LPS-stimulated RAW264.7 cells after 2, 4, and 6 h of incubation. **(b)** Statistical quantification of the red fluorescence intensities (MFI) corresponding to the time-dependent uptake in (a). **(c)** Comparative flow cytometric evaluation of cellular uptake for MB NPs, YW NPs, and MB@YW NPs in LPS-primed RAW264.7 cells. **(d)** Quantitative assessment of red fluorescence signals for the indicated nanoparticle formulations from (c).

**Figure S13. Comparative intracellular activation of various nanoparticle formulations in activated macrophages.** **(a)** CLSM images of LPS-stimulated RAW264.7 cells following a 6 h incubation with YW NPs, MB NPs, or MB@YW NPs. **(b, c)** Statistical quantification of the (b) HOCl-associated red and (c) ONOO⁻-associated blue fluorescence intensities across different treatment groups.

**Figure S14. Selective dual-channel fluorescence response of MB@YW NPs in RAW264.7 cells.** **(a)** Confocal images showing the activation of red and blue fluorescence in MB@YW NPs-treated cells upon exposure to exogenous (HOCl, SIN-1) or endogenous (LPS-induced) stimuli. **(b, c)** Corresponding quantitative analysis of (b) HOCl-associated red and (c) ONOO⁻-associated blue fluorescence intensities.

**Figure S15. *In vitro* spatiotemporal self-monitoring of MB@YW NPs delivery. (a)** CLSM images demonstrating the spatiotemporal activation of red and blue signals in control and LPS-activated RAW264.7 cells before and after photo-irradiation. **(b, c)** Corresponding quantitative analysis showing the (b) stabilization of red fluorescence and (c) attenuation of blue fluorescence following the photo-irradiation process.

**Figure S16. *In vitro* mitigation of oxidative stress and metabolic dysfunction by MB@YW NPs.** **(a)** Representative fluorescence images of intracellular ROS (DCFH-DA) and ATP (UDP-based sensor) in LPS-stimulated RAW264.7 macrophages across various treatment groups. **(b, c)** Corresponding quantitative analysis of (b) ROS-associated green fluorescence and (c) ATP-associated purple fluorescence intensities.

**Figure S17. Cytokine secretion profiles of RAW264.7 macrophages across different treatment groups.** **(a, b)** Quantitative ELISA analysis and the corresponding standard calibration curves for the pro-inflammatory cytokines TNF-α and IL-6. **(c, d)** Quantitative ELISA analysis and the corresponding standard calibration curves for the anti-inflammatory cytokines TGF-β and IL-10. Standard calibration curves were used to quantify the concentrations of cytokines. All biological samples were appropriately diluted to ensure their optical density (OD) values fell within the linear range of the corresponding standard curves.

**Figure S18. *In vivo* spatiotemporal self-monitoring of MB@YW NPs delivery. (a)** Schematic illustration of the experimental procedure for evaluating drug delivery after intravenous injection of MB@YW NPs and continuous localized irradiation in RA mice. **(b, c)** Corresponding quantitative analysis of the time-dependent (b) ONOO⁻-associated blue and (c) HOCl-associated red fluorescence intensities.

**Figure S19. Quantification of ROS and ATP levels in joint tissue homogenates. (a)** Schematic illustration of the fluorescence activation mechanisms for the ROS-responsive probe (DCFH-DA) and the ATP-responsive probe (UDP). **(b, c)** Relative fluorescence intensities of (b) ROS and (c) ATP in joint tissue homogenates from mice across different groups.

**Figure S20. Cytokine profiling in arthritic joint tissues across different treatment groups. (a, b)** Quantitative ELISA analysis and the corresponding standard calibration curves for the pro-inflammatory cytokines TNF-α and IL-6. **(c, d)** Quantitative ELISA analysis and the corresponding standard calibration curves for the anti-inflammatory cytokines TGF-β and IL-10. Standard calibration curves were used to quantify the concentrations of cytokines. All biological samples were appropriately diluted to ensure their OD values fell within the linear range of the corresponding standard curves.

**Figure S21. Histological examination (H&E staining) of major organs (heart, liver, spleen, lung, and kidney) collected from control mice and RA treated with PBS (RA group), YW NPs, MB NPs and MB@YW NPs, respectively.**

**Figure S22. Serum biochemistry analysis of hepatic and renal function markers for ALB, ALT, AST, CREA, UREA and UA levels in control group mice after treatment with PBS, YW NPs, MB NPs and MB@YW NPs.**

**Figure S23. Whole blood cell analysis of the control group mice treated with PBS, YW NPs, MB NPs and MB@YW NPs.**

**Figure S24. Venn diagram illustrating the overlap of differentially expressed genes (DEGs).** The diagram displays the intersection of DEGs identified from two comparisons: RA model vs. Control group, and MB@YW NPs-treated RA model vs. RA model group.

**Figure S25. Transcriptomic heatmap analysis of representative genes across key enriched immune and inflammatory signaling pathways.** Hierarchical clustering heatmaps display the relative expression profiles of differentially expressed genes (DEGs) enriched in the MAPK, Rheumatoid Arthritis, IL-17, JAK-STAT, and TNF signaling pathways across healthy control, untreated RA model, and MB@YW NPs-treated RAW264.7 macrophages.

**Figure S26. Transcriptomic insights into the therapeutic effects of MB@YW NPs.** Chord diagram depicting the enriched GO terms of differentially expressed genes (DEGs) between the RA model and the MB@YW NPs-treated RAW 264.7 cells.

**Figure S27. ^1^H NMR spectrum of MB-Cl in DMSO-*d*_6_.**

**Figure S28. ^1^H NMR spectrum of MB-BTD in DMSO-*d*_6_.**

**Figure S29. ^1^H NMR spectrum of MB-MTX in DMSO-*d*_6_.**

**Figure S30. ^13^C NMR spectrum of MB-MTX in DMSO-*d*_6_.**

**Figure S31. HRMS spectrum of MB-MTX.**

**Figure S32. ^1^H NMR spectrum of YW-Br in CDCl_3_.**

**Figure S33. ^1^H NMR spectrum of YW-TDP in CDCl_3_.**

**Figure S34. ^1^H NMR spectrum of YW-SSZ in CDCl_3_.**

**Figure S35. ^13^C NMR spectrum of YW-SSZ in DMSO-*d*_6_.**

**Figure S36. HRMS spectrum of YW-SSZ.**

**Figure S37. ^1^H NMR spectrum of PEG-Br in CDCl_3_.**

**Figure S38. ^1^H NMR spectrum of PEG-PND in CDCl_3_.**

**Figure S39. GPC chromatogram and molecular weight characterization of polymer PEG-PND in THF.**
